# A Novel Compound QO‐83 Alleviates Acute and Chronic Epileptic Seizures in Rodents by Modulating K_V_7 Channel Activity

**DOI:** 10.1111/cns.70334

**Published:** 2025-03-24

**Authors:** Xiangyu Wang, Yang Zhang, Hui Liu, Jiahao Wang, Boxuan Zhang, Tenghui He, Huiran Zhang, Zhumei Xiong, Xingang Liu, Jincan Li, Weidong Zhao, Xiao Liu, Wei Zhang, Le Yang, Qian Li, Hailin Zhang, Jinlong Qi, Qingzhong Jia

**Affiliations:** ^1^ Hebei Medical University, Postdoctoral Mobile Station of Basic Medical Hebei Medical University Shijiazhuang China; ^2^ College of Pharmacy Hebei Medical University Shijiazhuang China; ^3^ Department of Neurobiology Hebei Medical University Shijiazhuang China; ^4^ Department of Pharmacology Hebei Medical University Shijiazhuang China; ^5^ Department of Pharmacy Shijiazhuang Fifth Hospital Shijiazhuang China; ^6^ The Key Laboratory of Neural and Vascular Biology Ministry of Education, Hebei Medical University Shijiazhuang China; ^7^ Collaborative Innovation Center of Hebei Province for Mechanism, Diagnosis and Treatment of Neuropsychiatric Diseases Hebei Medical University Shijiazhuang China; ^8^ The Key Laboratory of New Drug Pharmacology and Toxicology Hebei Medical University Shijiazhuang China; ^9^ National Key Laboratory of New Pharmaceutical Preparations and Excipients Hebei Medical University Shijiazhuang China

**Keywords:** epilepsy, K_V_7 channel, neurological disorder, pyramidal neurons, QO‐83

## Abstract

**Aims:**

K_V_7 channels are promising targets for antiepileptic therapy. However, the classic K_V_7 channel opener retigabine has been withdrawn due to severe adverse reactions. We developed a novel K_V_7 channel opener, QO‐83, with good chemical stability and blood–brain barrier penetration, and sought to evaluate its K_V_7‐opening activity, antiepileptic effects, and mechanisms of action.

**Methods:**

We used patch‐clamp electrophysiology, electroencephalogram recordings, dynamic simulations, and various epilepsy models to investigate the mechanisms and antiepileptic activity of QO‐83.

**Results:**

Compound QO‐83 exhibits greater potency at K_V_7.2/7.3 channels compared to K_V_7.4 or K_V_7.5 channels. It shows superior efficacy for K_V_7.2 with voltage‐dependent opening than retigabine, with W236 identified as the key binding site for the K_V_7.2 channel. QO‐83 significantly inhibited epileptiform discharge and influenced hippocampal sEPSC and sIPSC amplitudes. QO‐83 has a more effective dose of 1 mg/kg in acute and chronic epilepsy models smaller than that of retigabine (10 mg/kg). The higher potency of QO‐83 may be attributed to its greater stability at the K_V_7.2 binding pocket compared to retigabine.

**Conclusion:**

QO‐83, as a newly developed Kv7.2 opener, has the advantages of stable properties, strong affinity, and high activity compared with retigabine, and is expected to become a new antiepileptic drug.

## Graphical abstract

QO‐83 has better opening effects on K_V_7.2 and K_V_7.2/7.3 channels, with W236 as the key site for its high binding free energy to K_V_7.2. By opening K_V_7 channels, regulating sIPSC and sEPSC amplitudes, and inhibiting epileptiform discharges, QO‐83 outperforms RTG in both acute and chronic epilepsy models.

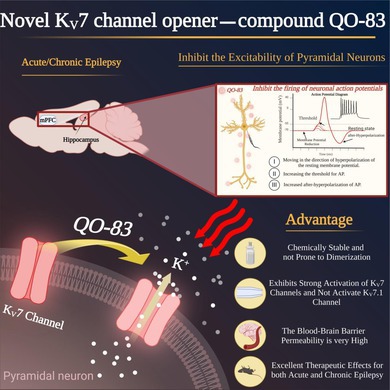

## 1 Introduction

Over 70 million individuals worldwide suffer from epilepsy, a condition characterized by abnormal neuronal discharge into the brain [1, 2]. In particular, hippocampal and cortical neurons frequently exhibit an abnormally excited state, with a relative imbalance between excitatory and inhibitory neurotransmitters. One major target in antiepileptic treatment is the K_V_7 channel (encoded by *KCNQ*), the opening of which causes hyperpolarization of the cell membrane, thereby inhibiting abnormally excited neurons and contributing to membrane stability [3, 4, 5, 6, 7]. K_V_7 channels can be divided into five subtypes (K_V_7.1–K_V_7.5), among which K_V_7.1 is found in the heart and subtypes K_V_7.2 to K_V_7.5 are widely distributed throughout the nervous system [8, 9].

*KCNQ2* and *KCNQ3* form heterotetrameric M channels that mediate neuronal M currents, and similarly, *KCNQ4* and *KCNQ5* contribute to M currents [10]. K_V_7.2 and K_V_7.2/7.3 are highly expressed in regions such as the cortex, hippocampus, amygdala, basal ganglia, and hypothalamus. K_V_7 channels (M‐channels) not only stabilize membrane potential through hyperpolarization but also mediate medium after‐hyperpolarization (mAHP) at depolarized membrane potentials, thereby modulating neuronal excitability and action potential frequency adaptation [11, 12]. M channels play roles in both presynaptic and postsynaptic processes, controlling presynaptic terminal membrane potential and the availability of volt‐gated Ca^2+^ channels, as well as regulating neurotransmitter release to stabilize neuronal membranes [13, 14]. Early‐onset epilepsy has been established to be associated with altered M‐current function attributable to congenital mutations in *KCNQ2* and *KCNQ3* [10, 15].

K_V_7 channel openers are considered highly promising agents for the treatment of epilepsy. The first K_V_7 ion channel opener (for K_V_7.2–7.5) developed for the treatment of epilepsy was retigabine (RTG) [16, 17, 18, 19], which was subsequently withdrawn from the market on account of the blue discoloration of different tissues after prolonged use [20]. A further drawback of RTG is its relatively low brain distribution (brain/plasma ratio of 0.16), which would accordingly necessitate the administration of higher doses, thereby reducing its safety margin and increasing the risk of off‐target effects [21]. Moreover, RTG can cause severe urinary retention and other central side effects [22, 23, 24].

Subsequent studies revealed that these adverse effects were attributable to the structure of RTG rather than to its target (K_V_7 channels) [25]. Given the multiple drawbacks of RTG, we designed and synthesized a novel compound, QO‐83, for which we secured U.S. and Chinese patents [26, 27]. At the time of writing, preclinical evaluations of the biological activity and safety of QO‐83 are nearing completion, and in this study, we measured the K_V_7‐targeting capacity of QO‐83 in vitro and in vivo and sought to elucidate the mechanisms underlying its antiepileptic effects. We believe that our findings could open new avenues for advancing epilepsy therapeutics.

## 2 Materials and Methods

### 2.1 Electrophysiology

Pipettes (1–6 MΩ) were filled with a solution of KCl (145 mM), MgCl_2_ (1 mM), HEPES (10 mM), and EDTA (5 mM). The pH was adjusted to 7.2 with NaOH. Electrophysiological measurements were obtained using an AXON Multiclamp 700 B patch‐clamp amplifier. The bath solution contained NaCl (144 mM), KCl (5 mM), MgCl_2_ (1 mM), CaCl_2_ (2 mM), and d‐glucose (10 mM), with the pH adjusted to 7.4 using NaOH. The protocol involved an increase in voltage from −120 mV to 40 mV in increments of 10 mV to evoke a series of K_V_7 currents (1000 ms per pulse) in the bath solution. For details on cell culture, please see Appendix S1 2.2.1.

### 2.2 Animals

Referencing prior studies that assessed the efficacy of epilepsy treatments, male rats or mice were consistently chosen for experiments [28, 29]. The animals used in this study were housed individually in separate cages and maintained under a 12‐h light/dark cycle at an ambient temperature of 22°C–25°C. Prior to the commencement of the study, the rats were acclimated for 7 days in the animal facility to ensure adaptation to the environment. All procedures related to feeding and experimentation strictly adhered to the guidelines set forth by the Animal Welfare and Ethics Committee of the Research Center for Safety Evaluation of New Drugs, Hebei Medical University.

### 2.3 Electrophysiological Recordings in mPFC and Hippocampal Slices

The experimental procedures involving Sprague–Dawley (SD) rats aged 8 weeks adhered strictly to the guidelines set forth by the Institutional Animal Care and Use Committee. Animals were anesthetized using isoflurane, followed by decapitation to obtain rat brains promptly. The brains were then immersed in a cold solution maintained at 4°C, containing sucrose (220 mM), KCl (2.5 mM), CaCl_2_ (2 mM), MgCl_2_ (4 mM), NaH_2_PO_4_ (1.25 mM), NaHCO_3_ (26 mM), and glucose (10 mM) at a pH range of 7.2–7.3. This solution provided optimal conditions for brain tissue preservation. Incubating isolated brain slices in Mg^2+^‐free cell solution is an in vitro method of replicating the abnormal neuronal firing associated with epilepsy. Under physiological conditions, Mg^2+^ blocks NMDA receptors and enhances the activity of gamma‐aminobutyric acid (GABA). Therefore, removing Mg^2+^ increases neuronal excitability [30].

For recording action potentials and resting membrane potentials, an internal solution was formulated with the following composition (in mM): K‐gluconate 140, NaCl 5, CaCl_2_ 1, MgCl_2_ 2, EGTA 11, HEPES 11, Mg‐ATP 2, and Na_2_‐GTP 0.5. Its pH was adjusted to 7.2–7.3. The electrode internal solution for recording sEPSCs and sIPSCs comprised (in mM): Cs‐gluconate 117, CsCl 11, HEPES 10, EGTA 11, CaCl_2_ 1, MgCl_2_ 1, Mg‐ATP 1, Na_2_‐GTP 4, and QX‐314 2; pH was adjusted to 7.2–7.3. Stimulation was applied in 20 pA increments every 500 ms, starting from 0 to 180 pA.

M current protocol: The amplitudes of M‐currents were measured as deactivating currents during 1000‐ms test pulses to −60 mV, starting from a holding potential of −30 mV. In current‐clamp experiments, the membrane potential was set to −70 mV. Testing was conducted after continuous perfusion for 5 min during the application of QO‐83, RTG, and XE991. During sIPSC recordings, the AMPA receptor antagonist CNQX (20 μM) and the NMDA receptor antagonist D‐AP5 (50 μM) were added to the freshly prepared Mg^2+^‐free ACSF solution. For sEPSC recordings, the GABA_A_ receptor antagonist bicuculline (50 μM) dissolved in dimethyl sulfoxide (DMSO) was added to the Mg^2+^‐free ACSF solution.

### 2.4 QO‐83 Against Pilocarpine‐Induced Status Epilepticus (SE) and Awake Electroencephalogram (EEG) Recordings

#### 2.4.1 EEG Electrode Implantation Surgery in a Pilocarpine‐Induced Acute Epilepsy Model

For animal experiments, we used adult male Sprague–Dawley (SD) rats weighing between 240 and 260 g. Isoflurane anesthesia was used for all operations. Rats were secured in a stereotaxic frame, and the anterior fontanelle was marked 3 mm posteriorly. Additionally, marks were made approximately 3 mm lateral to the midline on both sides to indicate the insertion points for the positive and negative electrodes. A ground electrode was placed subcutaneously in the occipital lobe to eliminate recording noise. Postoperatively, each animal was housed separately in a single cage and provided food and water ad libitum. After a 3‐day adaptation period, electroencephalograms (EEG) were recorded.

#### 2.4.2 Pilocarpine‐Induced Status Epilepticus (SE) Model

All the animals were randomly divided by weight, with male rats assigned to several groups: a control group, a pilocarpine (Pilo) model group, a QO‐83 group receiving a dosage of 1 mg/kg, and RTG groups receiving dosages of either 1 mg/kg or 10 mg/kg. The experimental procedure commenced with an intraperitoneal injection of lithium chloride (127 mg/kg). After 24 h, subcutaneous scopolamine methyl nitrate (1 mg/kg) was administered. Subsequently, the rats were intraperitoneally injected with either QO‐83, RTG, or 25% beta‐cyclodextrin. Twenty minutes later, status epilepticus (SE) was induced by administering pilocarpine (100 mg/kg, i.p.). SE was terminated 2 h later by administering 10% chloral hydrate.

The Pilo group, 2 mg/kg XE991 group, and 2 mg/kg XE991 + 1 mg/kg QO‐83 group received an intraperitoneal injection of lithium chloride (127 mg/kg) 24 h prior to testing. Following this, scopolamine methyl nitrate (1 mg/kg) was administered intraperitoneally, and after 10 min, each group was treated with 25% beta‐cyclodextrin, 2 mg/kg XE991, or 2 mg/kg XE991, respectively. After 15 min, 25% beta‐cyclodextrin, 25% beta‐cyclodextrin, or 1 mg/kg QO‐83 was administered. Finally, 25 min later, pilocarpine (100 mg/kg, i.p.) was administered to induce seizures.

#### 2.4.3 Seizure Severity Was Classified Using the Following Scale

Seizure severity was assessed using a standardized scale ranging from 1 to 5, adapted from previous research criteria. Stage 1 encompasses mouth and facial movements, including wet dog shakes. Stage 2 involves head nodding. Stage 3 is characterized by unilateral forelimb clonus. Stage 4 exhibits bilateral forelimb clonus and rearing behavior. Finally, stage 5 manifests as clonus of all limbs accompanied by falling.

### 2.5 Effects of QO‐83 on Acute and Chronic Epilepsy

#### 2.5.1 Maximal Electroshock Seizure (MES) Model

In this study, the effects of Compound QO‐83 on epilepsy were investigated using the Maximal Electroshock Seizure (MES) model. The experimental subjects were male Kunming mice (KM), each weighing between 18 and 25 g. QO‐83 was administered intraperitoneally (i.p.) 20 min before testing, and the compound was suspended in a 25% solution of beta‐cyclodextrin in distilled water. Various dose groups were established for administering Compound QO‐83, specifically at 0.5, 1, 1.5, 2, 2.5, and 3 mg/kg.

For comparative purposes, the positive control group received RTG at a dosage of 15 mg/kg (i.p.), while the control group was administered a solution containing only 25% beta‐cyclodextrin intraperitoneally. Electroconvulsions were induced using ear‐clip electrodes connected to a Rodent Shocker generator, which delivered a continuous wave output with the following parameters: stimulation current intensity of up to 150 V, stimulus duration of 20 ms, interval duration of 10 ms, wave number set at 60, and maximum stimulation current limited to 4 mA. Seizure activity was assessed based on the occurrence of tonic hind limb extension and evaluated according to predefined criteria.

#### 2.5.2 Metrazol Maximal Seizure (MMS) Model

For this experimental phase, male C57BL/6J mice weighing between 18 g and 25 g were randomly assigned to four groups based on their body weight. The QO‐83 group was divided into two subgroups, one receiving a dosage of 1 mg/kg (i.p.) and the other receiving a dosage of 3 mg/kg (i.p.). The RTG group received a dose of 15 mg/kg, while the model group was intraperitoneally administered an injection containing 25% β‐cyclodextrin at a volume of 0.1 mL per 10 g body weight.

PTZ (85 mg/kg, 0.1 mL per 10 g) was administered subcutaneously at the nape of the neck. Subsequently, mice were promptly placed in individual transparent observation chambers under quiet conditions. A stopwatch was utilized to measure seizure latency, defined as the time from PTZ injection to the onset of a generalized systemic clonic seizure and loss of the turning reflex. The number of animals experiencing seizures was manually recorded. Mice that did not display tonic convulsions during the subsequent 30‐min observation period were considered protected. Each mouse was observed for 30 min to compare seizure latency and the rate of protection against epileptic seizures among different groups.

#### 2.5.3 Penicillin (PNC) Induced Acute Epilepsy Model

In the penicillin (PNC) induced acute epilepsy model study, male SD rats weighing between 180 g and 250 g were utilized. The rats were randomly assigned to four groups: the PNC model group, the positive control RTG group, and two treatment groups receiving Compound QO‐83 at doses of 5 mg/kg (i.g.) and 10 mg/kg (i.g.). Each group consisted of 10 rats.

PNC was administered intraperitoneally at a dose of 8 million U/kg to induce epileptic seizures. The positive control group received an oral gavage of RTG at a dose of 40 mg/kg (i.g.), with a drug volume of 0.1 mL per 10 g body weight. In the Compound QO‐83 treatment groups, rats were orally administered the designated doses of Compound QO‐83, followed by an intraperitoneal injection of PNC after a 20‐min interval. The PNC model group received an oral gavage of a 0.5% CMC‐Na solution, followed by an intraperitoneal injection of PNC after a 20‐min interval. Similarly, in the RTG group, rats were first administered RTG orally and then injected with PNC intraperitoneally after a 20‐min interval. Seizure behaviors, including convulsions, limb jerks, and loss of postural control, were observed and analyzed in all groups.

#### 2.5.4 Preparation of Chronic PTZ‐Induced Epileptic Kindling Model

In the preparation of the chronic PTZ‐induced epileptic kindling model, male Wistar Institute rats weighing between 180 and 250 g were utilized. The rats were divided into the PTZ model group, the QO‐83 group (1 mg/kg), and the RTG group (10 mg/kg). During the modeling period, all groups received daily intraperitoneal injections of 0.2 mL/100 g between 9:00 and 15:00.

Each group was administered PTZ at a dose of 35 mg/kg for 30 days to induce chronic epileptic kindling. The RTG and QO‐83 groups were pretreated with their respective doses—10 mg/kg RTG and 1 mg/kg QO‐83—administered 20 min before the PTZ injection. The PTZ model group received an equivalent volume of 25% β‐CD as a control. Seizure severity was assessed using the Racine scale.

### 2.6 Molecular Dynamics Simulation

The protein structure of K_V_7.2 was obtained from the Protein Data Bank (https://www.rcsb.org/) using the PDB code 7CR2. Molecular docking was performed using the CDOCKER module in the DS 2020 software. Initial conformations of the QO‐83‐K_V_7.2 and RTG‐K_V_7.2 complexes were derived based on docking scores and conformational comparisons. The parameters for the K_V_7.2 protein were assigned using the Amber ff14SB force field, while the parameters for the ligand small molecules were assigned using the Amber GAFF force field. Partial charges for each atom of the ligand small molecules were initially calculated using Gaussian 09 at the B3LYP/6‐31G* level of theory and further refined using the RESP charge fitting program in Amber.

Molecular dynamics simulations were conducted using the sander program in Amber20. The simulated systems were optimized in a vacuum and placed in a TIP3P water box model. Periodic boundary conditions were applied during the simulation analysis. To maintain the system's neutrality, sodium or chloride ions were randomly added to the simulated systems. During the simulation process, the solute was initially restrained, and the system was optimized using 5000 steps of steepest descent minimization followed by 5000 steps of conjugate gradient minimization. Subsequently, the selected systems underwent 100 ns of molecular dynamics simulation in the NPT ensemble, with a simulation time step of 2 fs. The SHAKE algorithm was employed to constrain the bond lengths involving hydrogen atoms, and the Particle Mesh Ewald (PME) method was used to calculate long‐range electrostatic interactions. Finally, the root mean square deviation (RMSD) and binding/dissociation‐free energies of each system were calculated, and representative conformations were extracted.

### 2.7 Statistical Analysis

Normality tests on the data were conducted using nonparametric analyses for those that do not follow a normal distribution. Statistical differences between multiple groups (mean ± SEM) were compared using one‐ or two‐way ANOVA. Electrophysiological data were analyzed using SPSS version 21 and OriginPro (version 9.1.0). P Peak inward currents obtained from activation protocols were calculated using the following equation: G = I/(Vm‐EK), where G is conductance, I is peak inward current, Vm is instantaneous membrane potential, and EK is equilibrium potential for the sodium channel. Conductance data were normalized using maximum conductance and further fitted to the Boltzmann equation: $G=G_{\max}+\left(G_{\min}-G_{\max}\right)/\left(1+\exp\left[\left(V_{m}-V_{1/2}\right)/k\right]\right)$, where G is conductance, $V_{1/2}$ is midpoint of activation, and k is slope factor. The “Spectrum” module of LabChart was used for data analysis of EEG signals, including the calculation of power density and averaging power spectral density (PSD).

## 3 Results

### 3.1 QO‐83 Influences the Opening Activity of K_V_7.2‐K_V_7.5 Channels In Vitro

Details of the NMR spectra of synthesized QO‐83 are presented in Figures S1 and S2. Using Rb^+^ jet high‐throughput screening, we demonstrated that QO‐83 had a strong ability to open K_V_7.2/7.3 channels (EC_50_ = 0.08 ± 0.04 μM), although it showed poor selectivity for K_V_7.4 (EC_50_ = 0.84 ± 0.27 μM) (Figure S3). Importantly, QO‐83 did not trigger the opening of K_V_7.1 channels (Figure S3).

**FIGURE 1:**
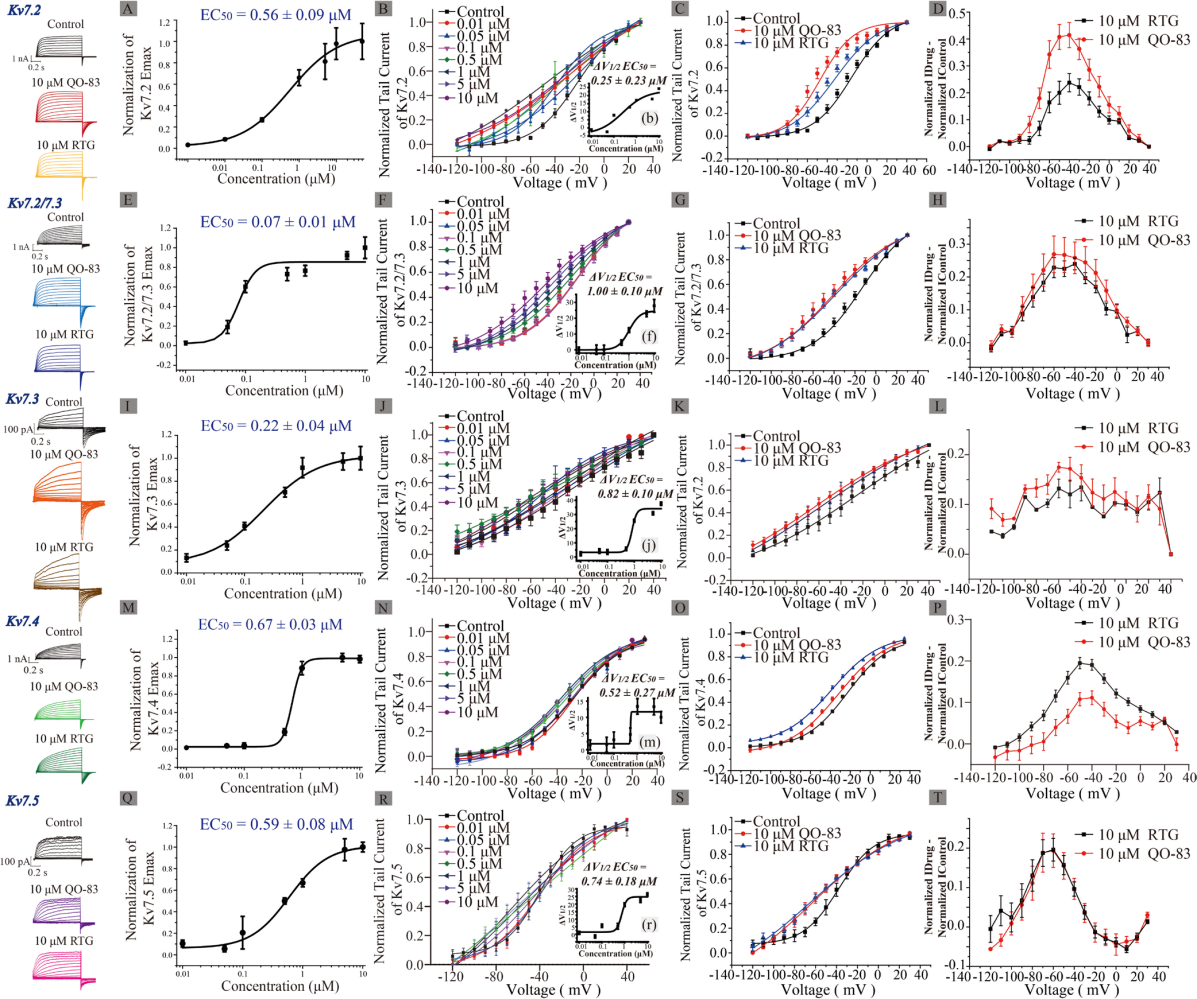
Kv7 channel subtype selectivity of compound QO‐83 and its activation curve. (A, E, I, M, and Q) EC_50_ of QO‐83 on K_V_7.2, K_V_7.2/7.3, K_V_7.3, K_V_7.4, and K_V_7.5 channels (Hill fitting; *n* = 7–12, 8, 8–10, 6–8, 6–8, respectively). (B, F, J, N, and R) Voltage‐dependent activation curves of normalized tail current of K_V_7.2–K_V_7.5 channels (Boltzmann fitting; *n* = 6–12). (B, F, J, N, and R) The dose–response curve of QO‐83 on the ΔV_1/2_ activation shift of K_V_7.2–K_V_7.5 channels (Hill fitting; *n* = 6–12). (C, G, K, O, and S) The effects of QO‐83 and RTG (both 10 μM) on K_V_7.2–K_V_7.5 activation curves (Boltzmann fitting; *n* = 6–12). (D, H, L, P, and T) The effects of compound QO‐83 on the Normalization I_Drug_—Normalization I_Control_ of K_V_7.2—K_V_7.5 channels at different voltages (*n* = 8–12).

As a further comparison of QO‐83 apparent affinity for each channel subtype, we used the manual patch clamp technique to obtain EC_50_ for opening K_V_7.2, K_V_7.2/7.3, K_V_7.3, K_V_7.4, and K_V_7.5 channels. Respectively, the results were 0.56 ± 0.09 μM (Figure 1A), 0.07 ± 0.01 μM (Figure 1E), 0.22 ± 0.04 μM (Figure 1I), 0.67 ± 0.03 μM (Figure 1M), and 0.59 ± 0.08 μM (Figure 1Q). These values accordingly indicate that, compared with other K_V_7 subtypes, QO‐83 shows a pronounced selectivity for K_V_7.2/7.3 channels.

We subsequently investigated the effects of QO‐83 on the voltage‐dependent activation of K_V_7 channel currents. Cells were perfused with different QO‐83 concentrations (0.01 μM, 0.05 μM, 0.1 μM, 0.5 μM, 1 μM, 5 μM, and 10 μM) for the recording. Corresponding EC_50_ values of ΔV_1/2_ were fitted as 0.25 ± 0.23 μM (K_V_7.2, Figure 1B), 1.00 ± 0.10 μM (K_V_7.2/7.3, Figure 1F), 0.82 ± 0.10 μM (K_V_7.3, Figure 1J), 0.52 ± 0.27 μM (K_V_7.4, Figure 1N), and 0.74 ± 0.18 μM (K_V_7.5, Figure 1R). QO‐83 shifts the V_1/2_ of K_V_7.2–K_V_7.5 channels in the hyperpolarizing direction. At a concentration of 10 μM, QO‐83 shifts the activation V1/2 of K_V_7.2, K_V_7.2/7.3, K_V_7.3, K_V_7.4, and K_V_7.5 channels by −26.48 mV, −27.79 mV, −37.67 mV, −10.01 mV, and −26.28 mV, respectively (Table S1). QO‐83 induces a concentration‐dependent shift of V_1/2_ in K_V_7.2–K_V_7.5 channels toward hyperpolarization, thereby promoting channel activation.

We have compared the tail current activation curves of 10 μM RTG and 10 μM QO‐83. The normalized current activation curve after drug perfusion was subtracted from that before drug perfusion. We observed that QO‐83 caused a greater hyperpolarization shift in the activation curve of K_V_7.2 than RTG (Figure 1B,C). Additionally, QO‐83 induced a greater increase in the tail current of K_V_7.2 than RTG (Figure 1D, Table S2). Shifts in the activation curves for K_V_7.3, K_V_7.2/7.3, and K_V_7.5 were not significantly different between QO‐83 and RTG, although V_1/2_ was more hyperpolarized under the former than the latter (Figure 1). In contrast, QO‐83 did not trigger greater activity than RTG in K_V_7.4 (Figure 1O,P). When stimulated with 10 mV, QO‐83 and RTG have no effect on the activation time (τ) of K_V_7.2–K_V_7.5. At −120 mV, QO‐83 and RTG both significantly extended the deactivation time (τ) of K_V_7.2–K_V_7.5 (Table S2). In summary, compared to RTG, QO‐83 exhibited greater selectivity and efficacy in opening K_V_7.2 and K_V_7.2/7.3 channels.

### 3.2 QO‐83 Modulates Neuronal Excitability and sIPSC/sEPSC Currents in Adult SD Rats In Vitro

**FIGURE 2:**
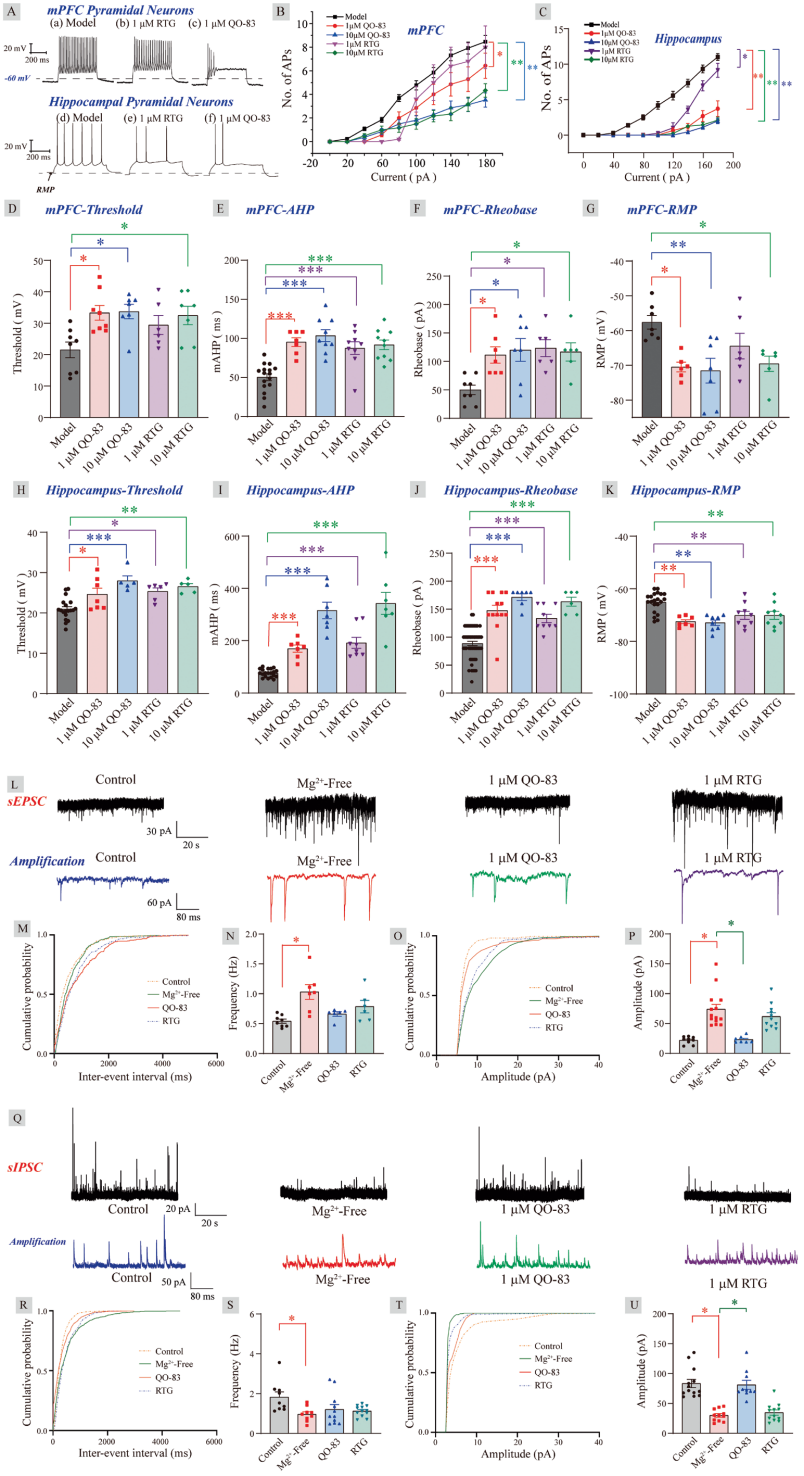
Effects of QO‐83 on hippocampal and cortical neuron excitability and sIPSC/sEPSC currents. (A) Typical curve depicting the effects of QO‐83 on action potential release in mPFC and hippocampal pyramidal neurons, induced by 180 pA current injection. (B, C) Summary graphs showing the inhibitory effects of QO‐83 on AP firing in mPFC and hippocampal pyramidal neurons. (D–K) Effects of QO‐83 on the threshold, AHP, rheobase, and RMP of mPFC and hippocampal pyramidal neurons stimulated by 180 pA currents. (L, Q) Traces of spontaneous sEPSC and sIPSC in hippocampal pyramidal neurons from different groups. (M, R) Cumulative probability analysis of sEPSC and sIPSC inter‐event interval curves per group. (N, S) Histogram showing the effect of QO‐83 on sEPSC and sIPSC frequencies. (O, T) Cumulative probability analysis of sEPSC and sIPSC amplitude curves per group. (P, U) Histogram showing the effect of QO‐83 on sEPSC and sIPSC amplitudes. *p < 0.05, **p < 0.01, ***p < 0.001; ANOVA with Bonferroni post hoc test.

Mg^2+^ blocks NMDA receptors and also enhances the activity of gamma‐aminobutyric acid (GABA). Therefore, removing Mg^2+^ from the medium increases neuronal excitability, which is a common method to observe abnormal electrical activity in neurons on brain slices [30]. At a concentration of 10 μM, both QO‐83 and RTG significantly inhibited AP firing (Figure 2B,C). However, at 1 μM, while QO‐83 still significantly reduced AP firing in mPFC and hippocampus pyramidal neurons, RTG had no significant effect (Figure 2A,B). Overall, QO‐83 demonstrated a stronger inhibitory effect on neuronal firing than RTG at lower concentrations.

Under the stimulation of 180 pA current, QO‐83 significantly increased the rheobase and threshold of mPFC and hippocampal pyramidal neurons, while significantly decreasing the RMP of these neurons (Figure 2D–K). Neuronal transmission depends on the frequency, mode, and timing of the pulse output, with after‐hyperpolarization (AHP) shaping all of these variables. QO‐83 significantly shifted the AHP of mPFC and hippocampal pyramidal neurons toward depolarization (as shown in Figure 2E,I). At 10 μM, RTG had the same effect as QO‐83, but at 1 μM, RTG had less impact on threshold and RMP than QO‐83. Interestingly, QO‐83 and RTG exhibited a stronger inhibitory effect on the excitability of hippocampal pyramidal neurons than on cortical pyramidal neurons, an outcome likely related to the high K_V_7.2 expression in the hippocampus. In conclusion, QO‐83 exerts membrane‐stabilizing effects by influencing the threshold, rheobase, resting membrane potential, and AHP.

Our results indicated that QO‐83 regulates ectopic discharge of hippocampal pyramidal neurons. In the context of neurological disorders, such as epilepsy, hippocampal neurons often exhibit abnormal excitability and a relative imbalance between excitatory and inhibitory neurotransmitters. Comparing the sEPSC and sIPSC provides a quantitative measure of the synaptic input balance, offering insights into the active inputs to these neurons. Without affecting sEPSC frequency, QO‐83 significantly inhibited sEPSC amplitude from 74.06 ± 8.08 pA to 23.20 ± 2.11 pA in the Mg^2+^‐free group (Figure 2L–P). Additionally, QO‐83 significantly reversed the effect of Mg^2+^‐free conditions, increasing sIPSC amplitude (with no effects on frequency) from 30.08 ± 3.02 pA to 81.15 ± 7.57 pA (Figure 2Q–U). In contrast, 1 μM RTG had no effect on either sEPSC or sIPSC.

### 3.3 QO‐83 Significantly Reverses Pilocarpine‐Induced Epileptic Status and Reduces Epileptoid Electroencephalogram Activity in Adult SD Rats

**FIGURE 3:**
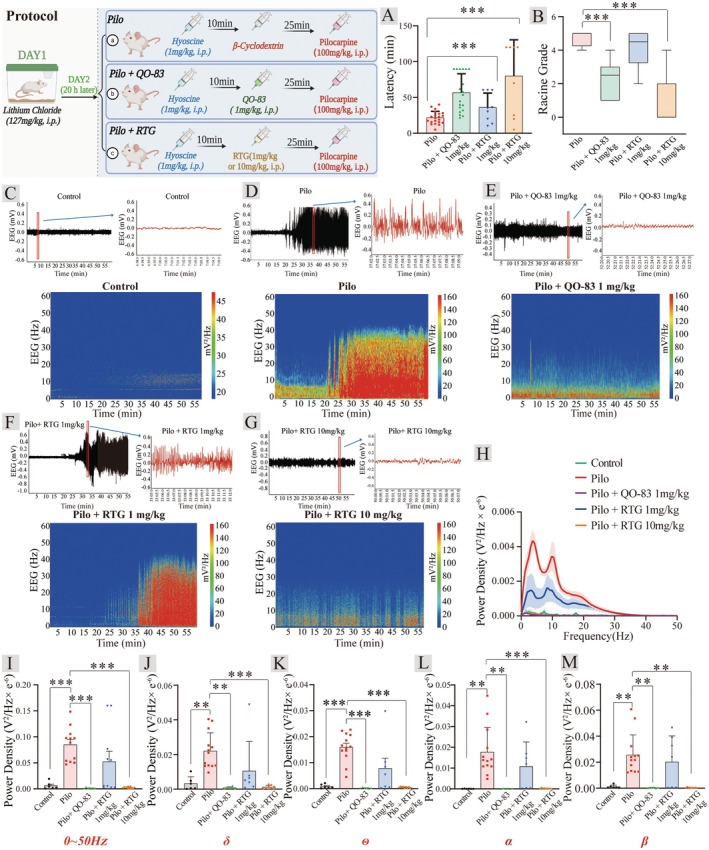
Effects of QO‐83 on EEG in pilocarpine‐treated rat models. (A) Latency of first grade III seizure (*n* = 8–21). (B) Histogram representing QO‐83's influence on seizure Racine grade in rats (*n* = 8–21). (C–G) Typical trace curves and spectrograms representing the effects of QO‐83 and RTG on electroencephalograms. (H) Power density‐frequency curve. (I) Bar chart of power density statistics for the frequency range of 0–50 Hz (*n* = 6–12). (J–M) Bar charts of power density for δ, θ, α, and β frequency bands (*n* = 6–12). (*p < 0.05, **p < 0.01, ***p < 0.001; The Shapiro–Wilk test was used to assess normality for all data; data presented in Figure 3B were analyzed using Kruskal–Wallis and Dunnett's tests, while others were analyzed using Bonferroni–ANOVA test statistics).

*KCNQ* channel openers have been evaluated as potential treatments for status epilepticus (SE), the most extreme form of epileptic seizure that can result in neuronal damage and dysfunction [31]. EEG was recorded in rats with systemic injection of pilocarpine to induce epileptic states (PILO‐SE). Recording brain activity while observing epileptic behavior revealed that QO‐83 significantly prolonged the latency of seizures from 21.64 ± 8.89 min to 56.79 ± 26.29 min (Figure 3A). QO‐83 reduced the seizure grade from 4.70 ± 0.12 to 2.33 ± 0.24 (Figure 3B). 1 mg/kg RTG could not rescue the epileptic behavior in mice, but 10 mg/kg RTG significantly prolonged the latency of seizures and reduced the seizure grade in the PILO model mice.

Pilocarpine‐induced increase in EEG activity involved the entire spectrum of frequencies analyzed (0.5–50 Hz), particularly in the higher range, such as the alpha (8–12 Hz) and beta (12–30 Hz) bands (Figure 3C–G). In pilocarpine‐treated rats, 1 mg/kg QO‐83 and 10 mg/kg RTG significantly inhibited the increase in EEG power spectral density until it returned to baseline across the entire frequency range (Figure 3H). The administration of QO‐83 resulted in a significant inhibition of PILO‐induced power enhancement in δ, θ, α, and β waves (Figure 3I–M). Notably, at 1 mg/kg, RTG did not exhibit effects equivalent to QO‐83 in either behavioral or epileptic EEG aspects. Higher RTG doses were required to achieve therapeutic efficacy. In summary, QO‐83 significantly suppressed epileptiform EEG activity, thereby preventing seizure and mitigating seizure behavior in pilot model rats.

### 3.4 Effects of the QO‐83 and XE991 Combination on KV7 Current In Vitro and Seizure Activity in PILO Model SD Rats

**FIGURE 4:**
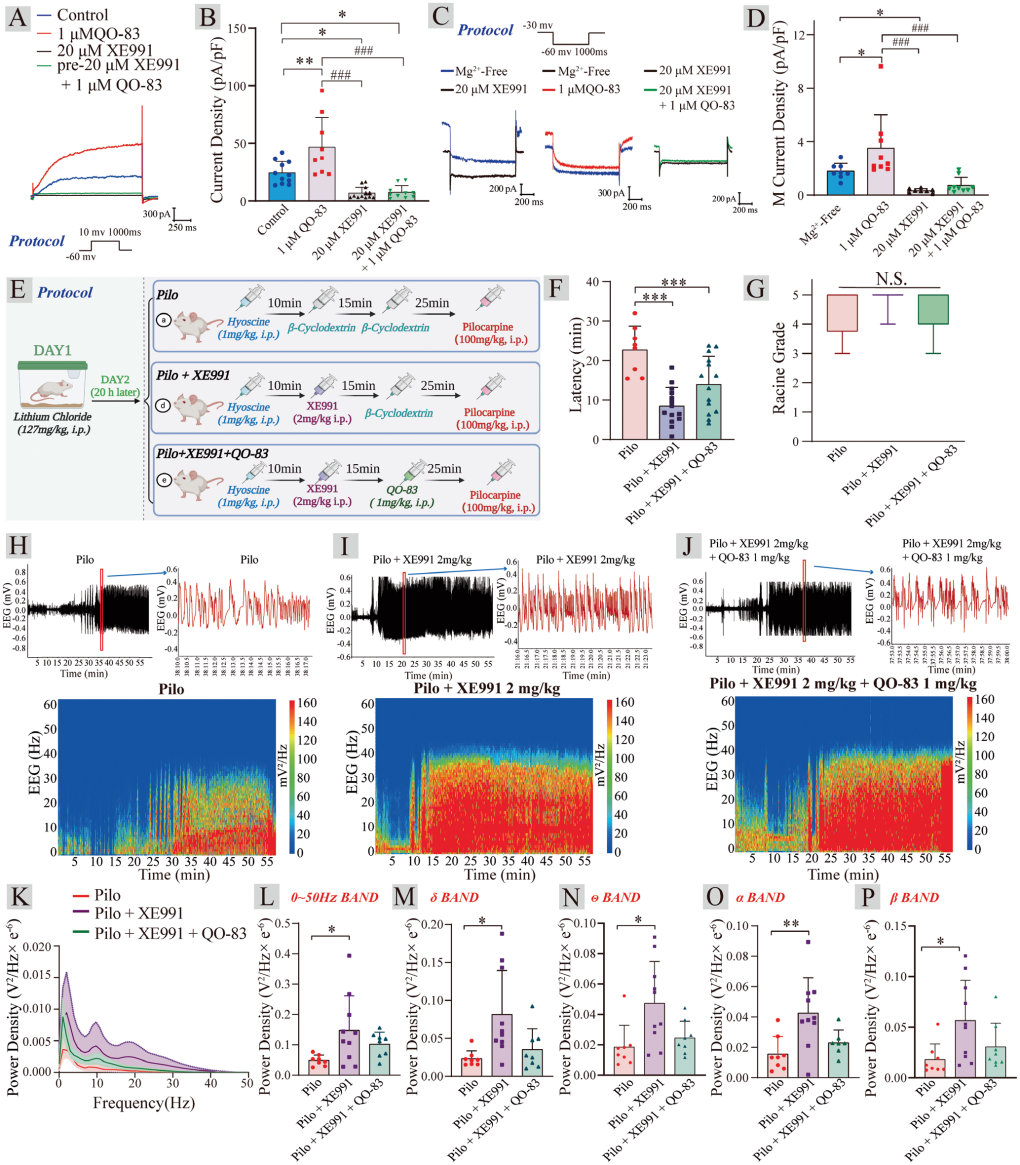
Effects of the QO‐83 and XE991 combination on K_V_7 current in vitro and seizure activity in PILO model SD rats. (A) Typical trace of the effect of QO‐83, RTG, XE991, and XE991 + QO‐83 on K_V_7.2 channel current. (B) Bar chart of the effect of QO‐83, RTG, XE991, and XE991 + QO‐83 on K_V_7.2 channel current density (*n* = 8–12). (C) Typical trace of the effect of QO‐83, RTG, XE991, and XE991 + QO‐83 on M channel current. (D) Bar chart of the effect of QO‐83, RTG, XE991, and XE991 + QO‐83 on M channel current density (*n* = 8–9). (E) Protocol for Pilo model and QO‐83, xe991 + QO‐83 combination treatment. (F) Latency of first grade III seizure (*n* = 8–10). (G) Histogram representing QO‐83's influence on seizure Racine grade in rats (*n* = 8–10). (H–J) Representative trace curves and spectrograms illustrating the effects of Pilo, XE991, and XE991 + QO‐83 on electroencephalograms. (K) Power density‐frequency curve. (L) Bar chart showing power density statistics for the 0–50 Hz frequency range (*n* = 8–10). (M–P) Bar charts of power density for δ, θ, α, and β frequency bands (*n* = 8–10). (*p < 0.05, **p < 0.01, ***p < 0.001; the Shapiro–Wilk test was used to assess normality for all data; data in panel (G) were analyzed using Kruskal–Wallis and Dunnett's tests, while other data were analyzed using Bonferroni‐ANOVA).

Based on the strong inhibitory effects of QO‐83 on neuronal excitability and epileptiform EEG activity, we investigate whether QO‐83 exerts its regulatory effects through the activation of K_V_7 channels. We examined the in vivo and in vitro activity of QO‐83 in combination with XE991. It was found that QO‐83 significantly increased the K_V_7.2 channel current (Figure 4A). The application of 20 μM XE991 significantly inhibited the increase in K_V_7.2 channel current induced by QO‐83 (Figure 4B). However, the co‐application of 20 μM XE991 + 1 μM QO‐83 did not further increase the K_V_7.2 channel current (Figure 4A,B). This indicates that XE991, by binding to the K_V_7.2 channel, prevents QO‐83 from interacting with the K_V_7.2 channel, thus blocking its activation effect. We further observed that QO‐83 significantly opened the M‐current in mPFC neurons (Figure 4C,D). After the perfusion of XE991, 20 μM XE991 + 1 μM QO‐83 failed to further enhance the M‐current, suggesting that XE991 blocks the interaction of QO‐83 with the M‐channel. These results indicate that the compound QO‐83 can significantly increase the K_V_7.2 channel and M‐current density, likely exerting its inhibitory effect on neuronal excitability through the activation of the M‐channel.

The administration of 2 mg/kg XE991 significantly reduced the seizure latency from 22.73 ± 6.03 min to 8.52 ± 4.73 min. However, co‐administration of 2 mg/kg XE991 with 1 mg/kg QO‐83 resulted in a seizure latency of 14.01 ± 7.10 min, which did not significantly prolong the latency (Figure 4E,F). No significant differences in seizure grades were seen between the model group, 2 mg/kg XE991, and 2 mg/kg XE991 + 1 mg/kg QO‐83 (Figure 4G). It is worth noting that, compared to the PILO group, 2 mg/kg XE991 significantly increased the power density within the 0–50 Hz frequency range in SD rats, as well as the power density in the δ, θ, α, and β bands (Figure 4H–P). Interestingly, when 2 mg/kg XE991 was combined with 1 mg/kg QO‐83, although there was a reduction in power density compared to 2 mg/kg XE991 alone, there was no statistically significant difference. These results suggest that the preferential administration of 2 mg/kg XE991 likely blocks K_V_7 channels, thereby hindering the anticonvulsant activity of QO‐83.

### 3.5 QO‐83 Effectively Alleviated Seizures in Both Acute Epilepsy Model Mice and Chronic Kindling Model Rats

**FIGURE 5:**
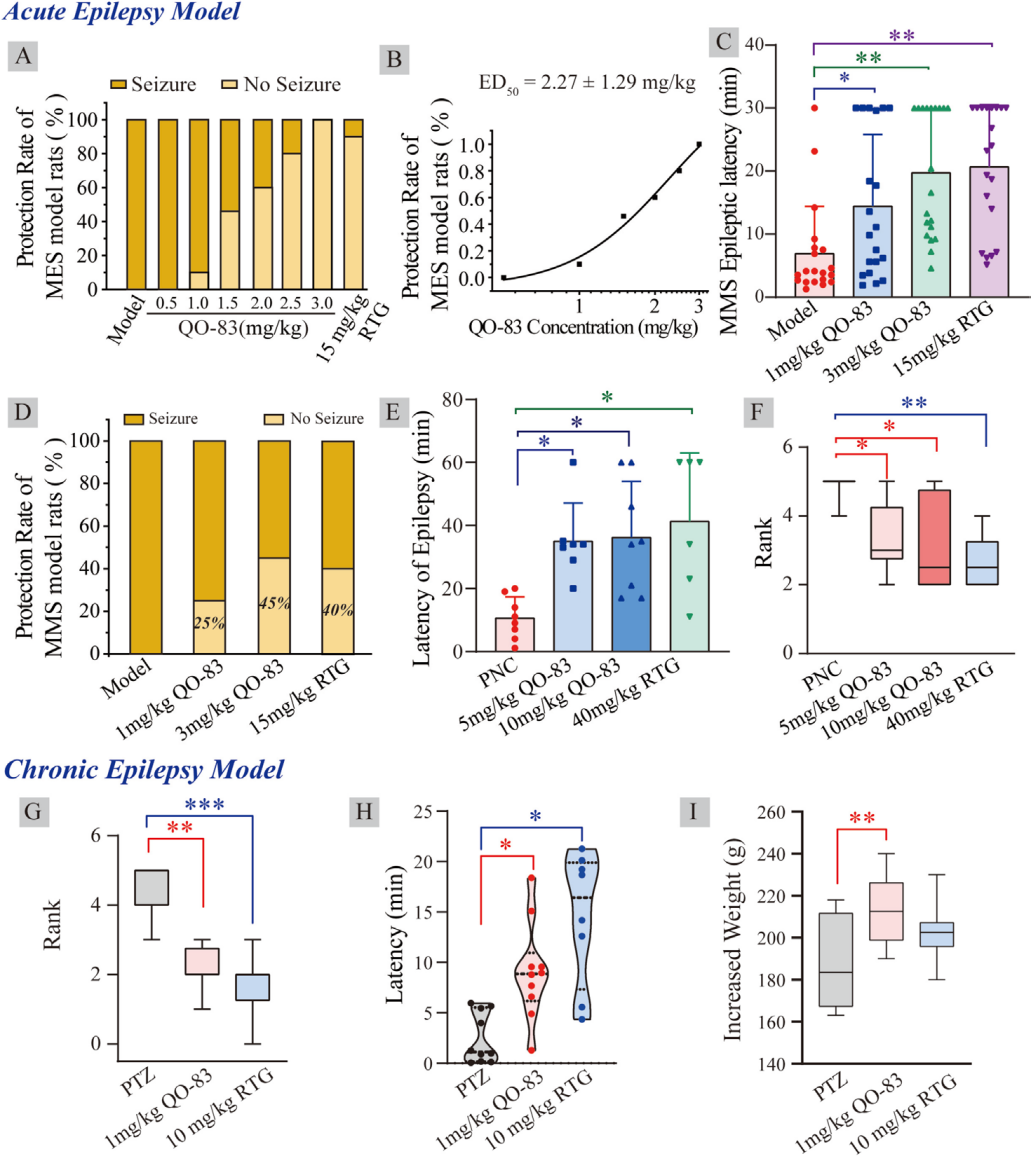
Therapeutic effect of QO‐83 on different epilepsy models. (A) Anticonvulsant protection rate of compound QO‐83 in the MES model (*n* = 10). (B) The dose‐effect curve of QO‐83 anticonvulsant protection rate (fitted to a logistic function, *n* = 10). (C) Epileptic latency of the MMS model (*n* = 20). (D) Anticonvulsant protection rate of QO‐83 in the MMS model (*n* = 20). (E) QO‐83 effect on the latency of the first grade III seizure in PNC model rats (*n* = 6–8). (F) QO‐83 effect on seizure grade scoring in PNC acute epileptic model rats (*n* = 6–8). (G) QO‐83 effect on seizure grade in PTZ‐induced chronic epileptic model rats (*n* = 8–10). (H) Latency of the first seizure to reach grade III in PTZ model rats (*n* = 8–10). (I) Histogram of body weight increase in rats per group. (*p < 0.05, **p < 0.01, ***p < 0.001; The Shapiro–Wilk test was used to assess normality for all data; Kruskal–Wallis with Dunn's post hoc test was applied for Figures F, G, and H, while ANOVA with Bonferroni post hoc test was used for Figures C, E, and I).

The maximal electroshock seizure (MES) model is widely used to screen anticonvulsant drugs. We tested QO‐83 at doses of 0, 1, 1.5, 2, 2.5, and 3 mg/kg; these treatments yielded electroshock protection rates of 0%, 10%, 50%, 60%, 80%, and 100% (Figure 5A). The anticonvulsant effect of QO‐83 was dose‐dependent, with an ED_50_ of 2.27 ± 1.29 mg/kg (Figure 5B). In contrast, treatment with 15 mg/kg of RTG led to 90% protection.

In the pentylenetetrazol maximal seizure (MMS) test, 85 mg/kg PTZ was subcutaneously injected into mice, inducing myoclonic jerks and generalized tonic seizures. QO‐83 at 1.0 mg/kg and 3.0 mg/kg significantly prolonged seizure latency from 6.92 ± 1.67 min to 14.45 ± 2.54 min and 19.43 ± 2.42 min, respectively. Under the same concentrations, RTG showed a similar effect on prolonging seizure latency (Figure 5C). Seizure protection rates of QO‐83 at 1 and 3 mg/kg were 25% and 45%, respectively, whereas 15 mg/kg RTG had a protection rate of 40% (Figure 5D).

In the penicillin(PNC)‐induced acute epilepsy model of SD rats, oral QO‐83 administration of 5 mg/kg and 10 mg/kg significantly prolonged seizure latency from 10.63 ± 2.39 min to 35.00 ± 4.61 min and 36.25 ± 6.29 min, respectively (Figure 5E). At a far higher concentration of 40 mg/kg, RTG also significantly increased seizure latency. Additionally, 5 mg/kg and 10 mg/kg QO‐83 reduced seizure grades to 3.33 ± 0.42 and 3.12 ± 0.47, respectively, while RTG reduced it to 2.66 ± 0.33 (Figure 5F).

On the 30th day of continuous administration of 35 mg/kg PTZ, changes in seizure parameters were observed in each group of rats. QO‐83 at 1 mg/kg and 10 mg/kg RTG significantly reduced seizure grades from 4.68 ± 0.62 to 2.12 ± 0.22 and 1.75 ± 0.31, respectively (Figure 5G). QO‐83 and RTG can both significantly prolong the latency period in the PTZ chronic kindling model in rats (Figure 5H). Furthermore, QO‐83 at 1 mg/kg significantly increased rat body weight, but RTG did not (Figure 5I).

In summary, QO‐83 has demonstrated potent anti‐convulsant effects in both acute and chronic epilepsy models. Notably, while RTG is administered at a dose 10 times higher than that of QO‐83, the latter achieves significant therapeutic effects at a much lower dose.

### 3.6 Comparison of the Binding Mechanisms of QO‐83 and RTG

**FIGURE 6:**
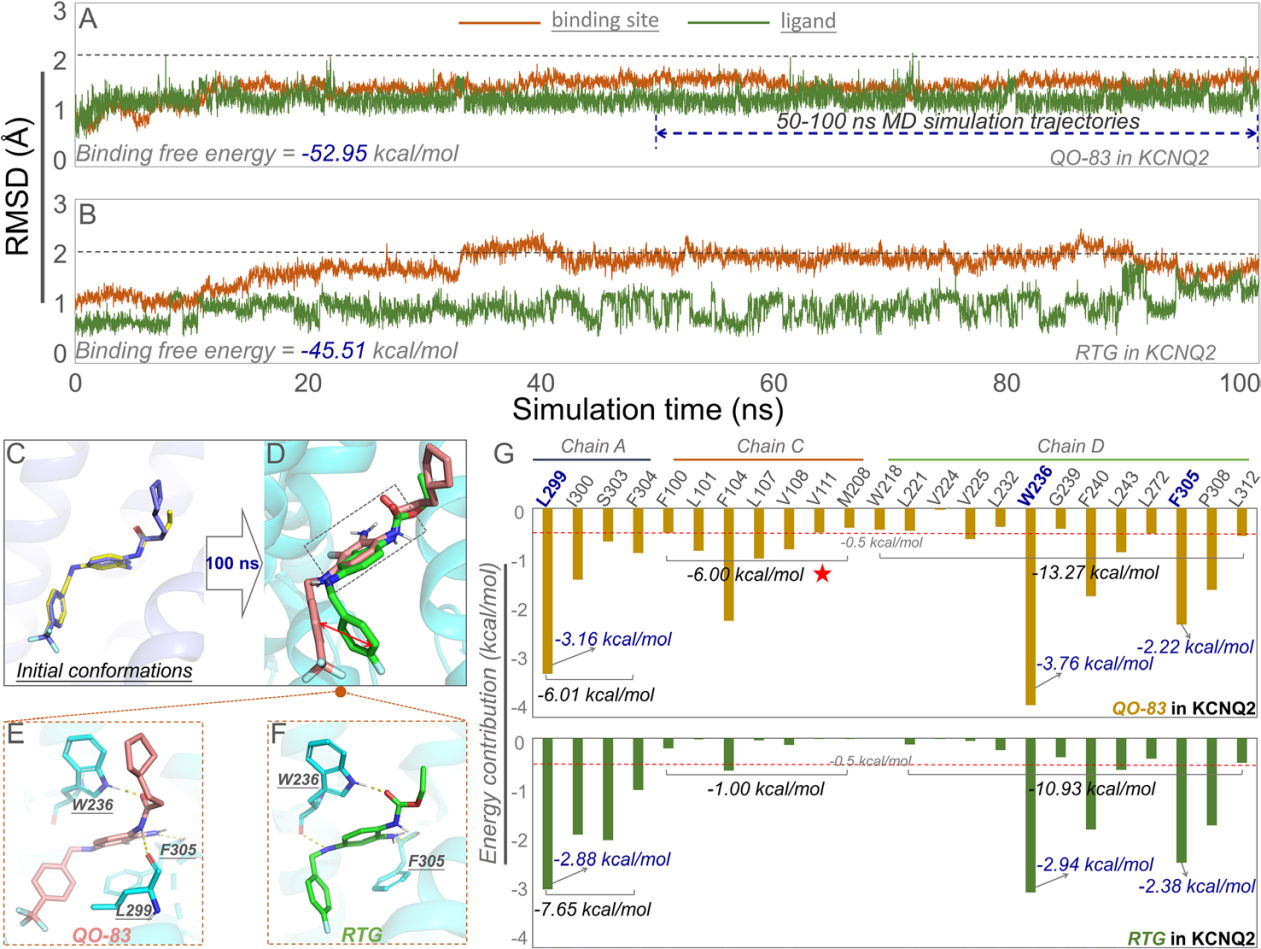
QO‐83 molecular dynamics simulations in the K_V_7.2 protein system. (A) Temporal variation in RMSD value and binding free energy of the QO‐83‐K_V_7.2 complex. (B) Temporal variation in RMSD value and binding free energy of the RTG‐K_V_7.2 complex. (C–F) Schematic of representative QO‐83 and RTG conformations when bound to K_V_7.2 and their interactions with key amino acids. (G) Schematic representation of residue energy decomposition in simulated QO‐83 and RTG binding to K_V_7.2.

To compare the binding modes of QO‐83 and RTG in K_V_7.2, molecular dynamics (MD) simulations lasting 100 ns were performed. The QO‐83/K_V_7.2 complex remained relatively stable throughout the simulation (Figure 6). The RMSD values for the residues at the binding site were consistently within 2 Å. Furthermore, QO‐83 exhibited relatively low fluctuations compared to RTG (Figures 6A and 5B). Next, we calculated the binding free energies of both compounds using MD trajectories for the last 50 ns. QO‐83 had greater target affinity with K_V_7.2 (−52.95 kcal/mol) than RTG (−45.51 kcal/mol), indicating a strong interaction between QO‐83 and K_V_7.2 active residues.

We compared the binding conformations of QO‐83 and RTG to K_V_7.2. Superimposition of the initial docking poses clearly revealed a high spatial overlap between QO‐83 and RTG (Figure 6C). The amount of space overlap differed between distinct QO‐83 and RTG conformations (Figure 6D). Moreover, the trifluoromethyl‐substituted benzene ring on QO‐83 differed from the corresponding benzene ring on RTG; however, other benzene rings significantly overlapped between the two compounds. Both QO‐83 and RTG formed hydrogen bonds with W236 and F305 (Figure 6E,F).

Repositioning identified residues revealed that amino acids in chains A, C, and D were the main energy contributors to binding. Residues on chain A contributed −6.01 kcal/mol and −7.65 kcal/mol of energy to QO‐83 and RTG, respectively, while residues on chain D contributed −13.27 kcal/mol and −10.93 kcal/mol (Figure 6G). Notably, W236 was a significant contributor to the binding energies of both QO‐83 and RTG. Chain C differed significantly in energy contributions to QO‐83 and RTG (6.00 kcal/mol vs. 1.00 kcal/mol), explaining the former's stronger binding affinity to K_V_7.2. The chain C residues (F104, L107, and V108) we identified offer valuable insights for future receptor‐based drug design.

## 4 Discussion

In recent years, studies have demonstrated the commendable efficacy of RTG in treating epilepsy, traumatic brain injury, and other neurological disorders [32, 33]. Building upon this remarkable effectiveness and aiming to address the issue of side effects avoidance, we have developed QO‐83. QO‐83 demonstrated enhanced potency in activating K_V_7.2, both in terms of voltage activation and current opening levels. However, previous reports indicate that RTG exhibits the strongest opening ability on the K_V_7.3 channel, highlighting a significant difference in subtype selectivity compared to our QO‐83 [34, 35, 36, 37]. Notably, although QO‐83 and RTG share the same key binding site (W236), the QO‐83‐K_V_7.2 complex exhibits greater stability and stronger interactions than the RTG‐Kv7.2 (Figure 6 and Figure S5) [36]. The advantage of QO‐83 may be attributed to its enhanced stability in binding to the C chain, particularly at F104, while maintaining efficient binding to sites such as W236 (Figure 6 and Figure S5B). This also underlies its lower EC_50_ for channel activation on K_V_7.2–7.5, indicating greater bioactivity than RTG, as discussed above.

As a small molecule drug targeting the central nervous system, blood–brain barrier permeability is crucial. In previous reports, the brain‐to‐plasma drug concentration ratio for RTG was 0.16 [37]. In contrast, 15 min after intraperitoneal administration, our QO‐83 exhibited a brain‐to‐plasma drug concentration ratio of 343.67%, demonstrating a significant improvement (Figure 5). The neuronal circuits activated during a seizure typically spread from the hippocampus, a common site of seizure onset, to the cortex [38]. Therefore, when observing various parameters affecting hippocampal and cortical neurons, we found that at the same concentration of 1 μM, RTG could not regulate the threshold for triggering action potentials and membrane potential, which are two crucial parameters. This indicates that QO‐83 has a lower effective dose and better efficacy compared to RTG. Following treatment with 20 μM XE991, QO‐83 failed to restore the M‐current, consistent with behavioral observations where pre‐administration of 2 mg/kg XE991 abolished the anticonvulsant effects of QO‐83. These findings strongly indicate that the anticonvulsant activity of QO‐83 is predominantly mediated through its activation of M‐channels.

Interestingly, QO‐83 does not affect the frequency of sIPSCs or sEPSCs but modulates their amplitude. Previous studies have shown that low concentrations of RTG do not impact GABA_A_ receptor currents, while blocking K_V_7.2/7.3 (M) channels with XE991 increases EPSP amplitude and depolarizes the RMP [39, 40]. This suggests that K_V_7.2/7.3 (M) channels regulate the intrinsic excitability and synaptic responses of PFC pyramidal neurons [40]. 10 μM RTG can enhance GABA_A_ receptor currents through α1β2δ, α4β2δ, α4β3δ, and α6β2δ receptors. In our experiments with a 1 μM concentration, which is relatively low, this could be the main reason why RTG does not affect the amplitude and frequency of sIPSCs [39]. However, it is worth noting that at a concentration of 1 μM, QO‐83 demonstrated suppression of sIPSC amplitude, indicating that QO‐83 has superior activity compared to RTG.

During structural optimization, we tested the chemical stability of QO‐83 to determine whether it performs better than the notoriously unstable RTG. Even after 1 month of storage, QO‐83 did not change in solution color. In contrast, RTG easily formed dimers with melanin, the primary cause of the blue‐purple precipitate common to this drug [41]. We did not observe any link between QO‐83 dipole formation and pigment development (Figures S6–S9), demonstrating good chemical stability. Due to the excellent biological activity observed in the in vitro and neuronal studies, the anticonvulsant doses of QO‐83 used in acute and chronic epilepsy models are significantly lower than those of RTG, indicating that QO‐83 is more effective than RTG. Additionally, no hepatotoxicity was observed with QO‐83 while exerting its anti‐convulsant activity (Figure S10). Overall, QO‐83 was more stable and safer than RTG. We recommend further development in the direction of QO‐83 and other K_V_7 channel openers.

## 5 Conclusions

Novel compound QO‐83 demonstrated excellent capacity to open K_V_7 channels, especially K_V_7.2 and K_V_7.2/7.3. Through opening these channels, QO‐83 inhibits neuronal excitability, thereby reducing epileptiform EEG activity. QO‐83 also modulates hippocampal pyramidal neurons by affecting sIPSC and sEPSC amplitudes. These mechanisms underlie the therapeutic efficacy of QO‐83 against acute and chronic epilepsy. Simultaneously, QO‐83 exhibited improved chemical stability and a higher BBB passage rate than RTG. The discovery of QO‐83 provides new insights and a promising drug candidate for epilepsy treatment targeting the K_V_7.2 channel.

## Author Contributions

Conception or design of the study: Hailin Zhang, Jinlong Qi, and Qingzhong Jia; data collection: Xiangyu Wang, Yang Zhang, Jiahao Wang, Huiran Zhang, and Zhumei Xiong; data analysis and interpretation: Jiahao Wang, Hui Liu, Le Yang, Wei Zhang, Xiao Liu, Jincan Li, Tenghui He, and Weidong Zhao; drafting the article: Xiangyu Wang, Boxuan Zhang, Qian Li, and Xingang Liu; critical revision of the article: Jinlong Qi and Qingzhong Jia. All authors approved the final version of the manuscript.

## Ethics Statement

The experiments on mice and rats were conducted in accordance with the guidelines of the Animal Welfare and Ethics Committee of the New Drug Safety Evaluation Research Center at Hebei Medical University, under the study number IACUC‐Hebmu‐PD‐2022032. The animal use license for the New Drug Safety Evaluation Research Center at Hebei Medical University is SYXK(JI)‐2018‐005. All methods were performed in accordance with the Guide for the Care and Use of Laboratory Animals.

## Consent

The authors have nothing to report.

## Conflicts of Interest

The authors declare no conflicts of interest.

## Supporting information


Appendix S1.

## Data Availability

The data that support the findings of this study are available from the corresponding author upon reasonable request.
