# Supplementary material for: A Novel Compound QO‐83 Alleviates Acute and Chronic Epileptic Seizures in Rodents by Modulating KV7 Channel Activity
Source: CNS Neurosci Ther. 2025 Mar 24;31(3):e70334. doi: 10.1111/cns.70334 (PMC11931445; doi:10.1111/cns.70334)
Supplement: Supplementary file 1 — Appendix S1. [file CNS-31-e70334-s001.docx]

**Supporting Information**

**1 Result**

***1.1 Preparation of Compound QO-83 in Synthesis***

Intermediate compound 2 (**Figure S1**) was synthesized by reacting 2, 3-difluoro-6-nitroaniline with (4-(trifluoromethyl)phenyl) methanamine in dimethyl sulfoxide (DMSO). The reaction utilized 1.2 equivalents of triethylamine and a catalytic amount of iodine. The reaction mixture was heated to 120°C and maintained at this temperature for 24 hours. The resultant intermediate 2 was subsequently reacted with zinc powder and saturated ammonium chloride in methanol. The reaction was stirred at room temperature for 5 hours, yielding 3-fluoro-N4-(4-(trifluoromethyl) benzyl) benzene-1,2,4-triamine (intermediate compound 1, Figure S1). Intermediate compounds 1 and 3-cyclopentylpropanoic acid were combined in a 1:1 molar ratio and added to dried methylene chloride. The mixture was then treated with 2 equivalents of 1-ethyl-3-(3-dimethylaminopropyl) carbodiimide (EDC) and a catalytic amount of 4-dimethylaminopyridine (DMAP). The reaction was stirred at room temperature for 24 hours. After completion, the methylene chloride layer was washed with water, adjusting the pH to 4-5. The organic layer was separated and concentrated under reduced pressure. The resulting solid was washed with 100% ethanol to yield compound QO-83 as a white solid. Compound QO-83 was characterized as a white crystalline solid with a melting point of 203-205°C. The purity was determined to be greater than 99% by HPLC-DAD, and the overall yield was approximately 80%.


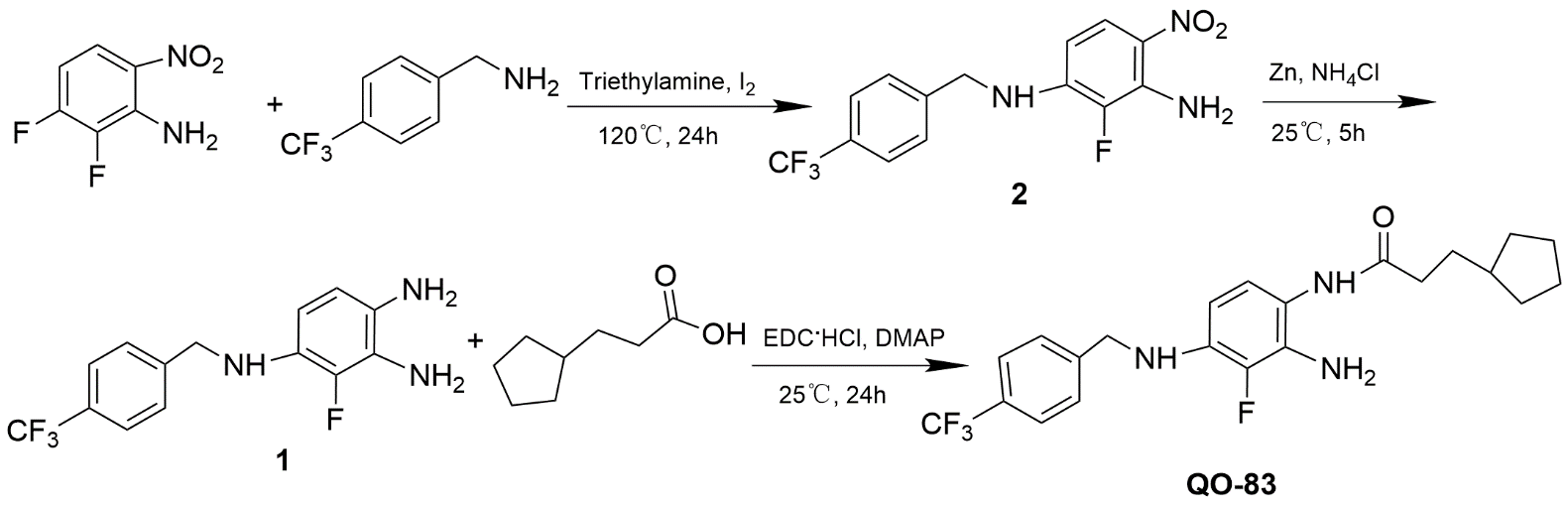
**Figure S1: Synthetic scheme of compound QO-83**

***1.2  ^1^H-NMR and ^13^C-NMR information of QO-83***

QO-83 ESI-HRMS (m/z): 424.1938 [M +H]^+^. ^1^H-NMR (500 MHz, DMSO-d6) &: 1.46 ~ 1.76 (m, 9H, Cyclopentyl-H), 1.08(d, 2H, -C = OCH_2_CH_2_), 2.25 (t, 2H, C = OCH_2_CH_2_), 4.38 (d, 2H, CH_2_NH), 4.58 (s, 2H, NH_2_), 5.77 (t, 1H, CH_2_NH), 6.02 (t, 1H, Ph-H), 6.57 (d, 1H, Ph-H), 7.54 (d, 2H, Ph-H), 7.66 (d, 2H, Ph-H), 8.98 (s, 1H, C = ONH). ^13^C-NMR (125 MHz, DMSO-d6) &: 25.19 (Cyclopentyl CH_2_), 32.52 (Cyclopentyl CH_2_), 32.08 (CH_2_), 35.41 (COCH_2_), 46.16 (Cyclopentyl CH), 100.06 (CH_2_NH), 127.34 (CF_3_), 171.98 (C = O), 146.22 (C), 141.61 (Ph-C), 139.79 (Ph-C), 134.40 (Ph-C), 131.42 (Ph-C), 127.90 (Ph-C), 125.96 (Ph-C), 125.56 (Ph-C), 123.80 (Ph-C), 121.51 (Ph-C), 115.57 (Ph-C), as shown in **Figure S2**.


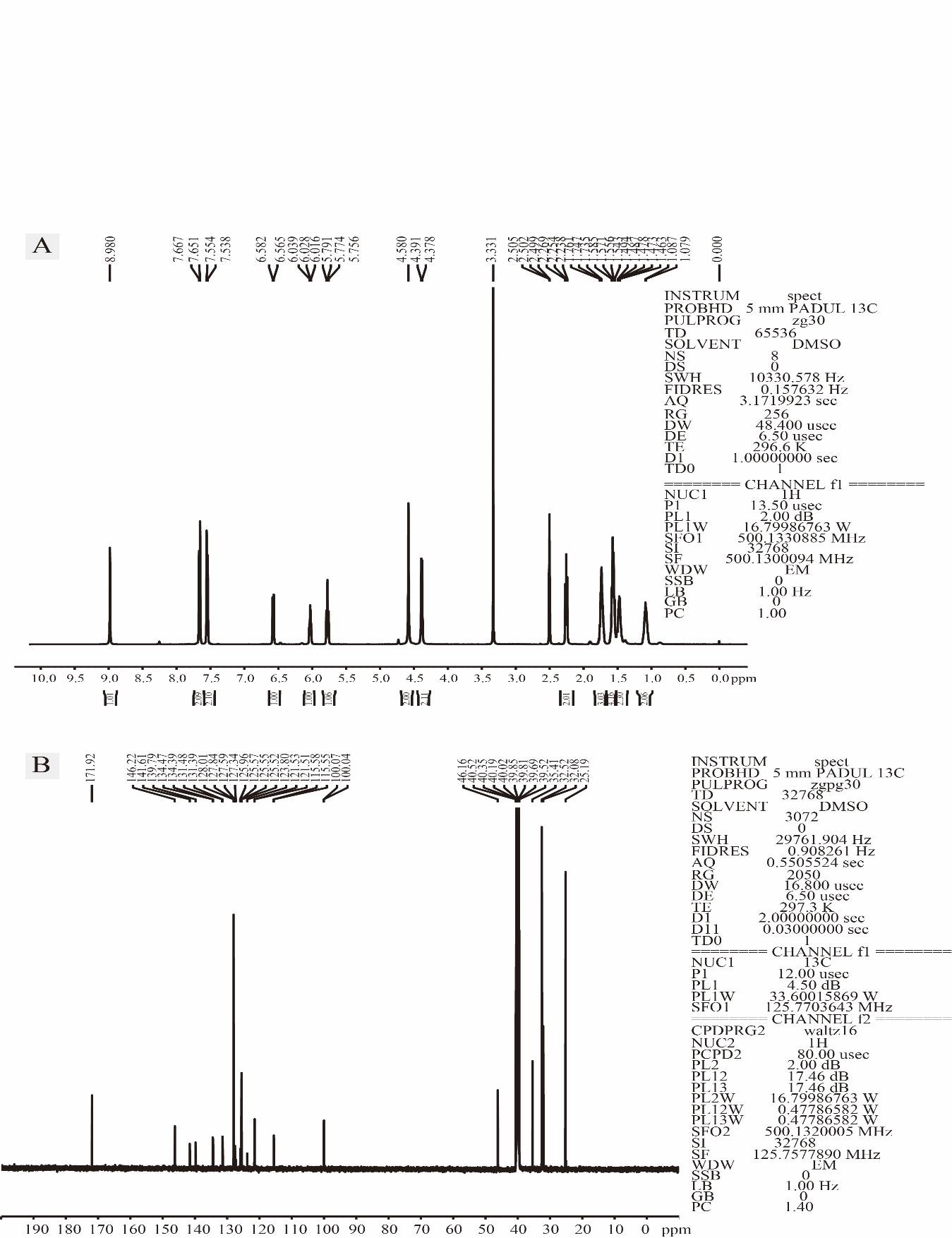
**Figure S2:** **The ^1^H-NMR and ^13^C-NMR spectrum of compound QO-83.** (A) The ^1^H-NMR spectrum of compound QO-83. (B) The ^13^C-NMR spectrum of compound QO-83.

***1.3 The Activation Effect of QO-83 on the K_V_7 Channel was Preliminary verified by High-throughput Screening.***

The activation effect of QO-83 on the K_V_7 channel was preliminarily verified using high-throughput screening methodologies. Utilizing Rb^+^ efflux high-throughput screening technology, the EC_50_ of RTG for activation of K_V_7.2/7.3 channels was determined to be 0.18 ± 0.07 μM, whereas QO-83 exhibited an EC_50_ of 0.08 ± 0.004 μM, indicating superior potency of QO-83 compared to RTG, with both compounds displaying similar efficacy (**Figure S3A**). Furthermore, the EC_50_ values for RTG and QO-83-induced activation of K_V_7.4 channels were determined as 1.38 ± 0.31 μM and 0.84 ± 0.27 μM, respectively (**Figure S3B**). These results highlight the relative selectivity and enhanced potency of QO-83 in comparison to RTG.

Furthermore, the K_V_7.1 channel, which co-expresses with the auxiliary subunit KCNE1 to form slowly activated delayed rectifier potassium currents (IKs) during cardiac repolarization, was evaluated using the Rb^+^ efflux high-throughput screening technique to determine the opening activity of QO-83 on the *KCNQ*1/E1 channel. Even at a concentration of 100 μM, RTG did not affect the K_V_7.1/KCNE1 channel, nor did QO-83 at the same concentration (**Figure S3C**). The experiments above demonstrate that QO-83 does not activate the K_V_7.1 channel at effective concentrations for K_V_7.2/7.3 and Kv7.4 channels. This implies that therapeutic doses of QO-83 do not impact cardiac function through the activation of K_V_7.1 channels.

**
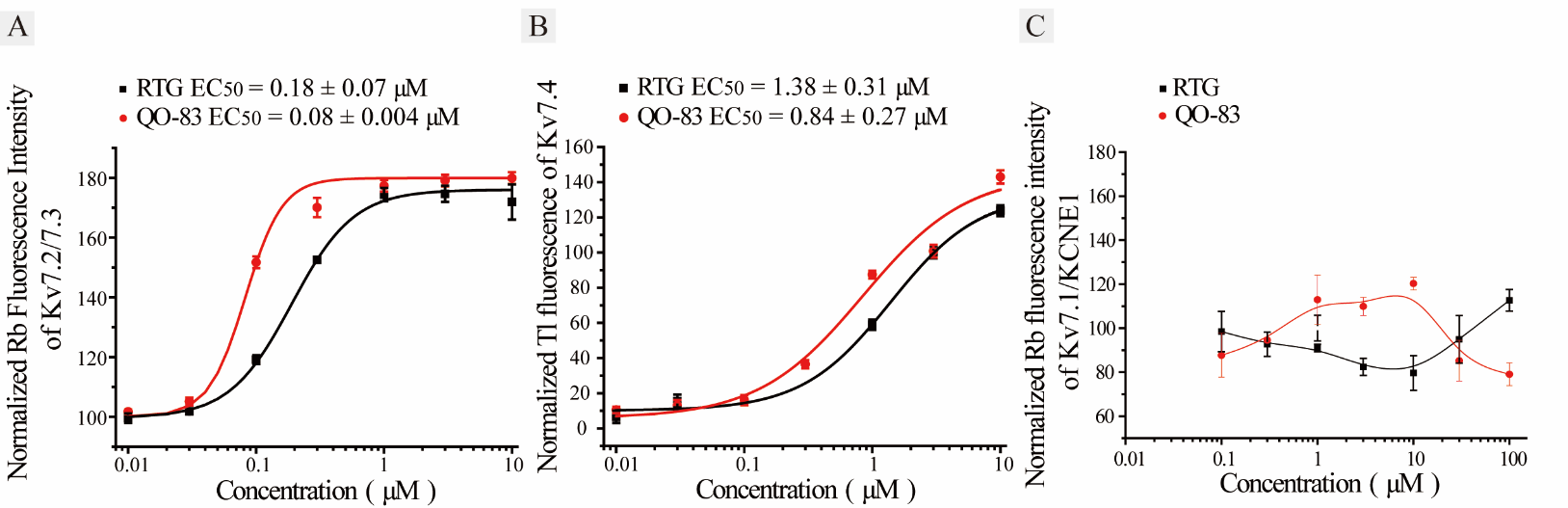
****Figure S3:** **The activation effect of QO-83 on the K_V_7 channels was preliminarily verified by high-throughput screening.** (A)High-throughput screening for K_V_7.2/7.3 stable expressed in CHO cells (n = 6). (B)High-throughput screening for K_V_7.4 stable expressed in HEK293 cells (n = 6). (C) High-throughput screening for *KCNQ*1/E1 transient expression in HEK293 cells (n = 6).

***1.4 Blood Brain Barrier Permeability of QO-83***

The blood-brain barrier permeability of QO-83 was assessed using HPLC-MS/MS analysis to determine the concentrations of the compound in various tissues at different time points. Following 15 minutes of gastric administration, QO-83 was rapidly and extensively distributed across multiple tissues. At the 1-hour mark, the concentration of QO-83 increased in all tissues examined. Specifically, after 15 minutes of administration, the brain tissue content of QO-83 was measured at 94.58 ± 26.48 ng/g, while the plasma drug content was 27.52 ± 25.46 ng/g, resulting in a brain-to-plasma concentration ratio of 343.67%. One-hour post-administration, the brain tissue content of QO-83 increased to 172.25 ± 75.13 ng/g, with a plasma drug content of 102.11 ± 96.64 ng/g, yielding a brain-to-plasma concentration ratio of 168.73% (**Figure S4**).

In summary, QO-83 demonstrated rapid and extensive distribution throughout the rat body, with significantly higher drug concentrations observed in brain tissue compared to plasma levels at concurrent time points. These findings suggest that QO-83 readily crosses the blood-brain barrier, indicating its potential as a therapeutic agent for central nervous system disorders.


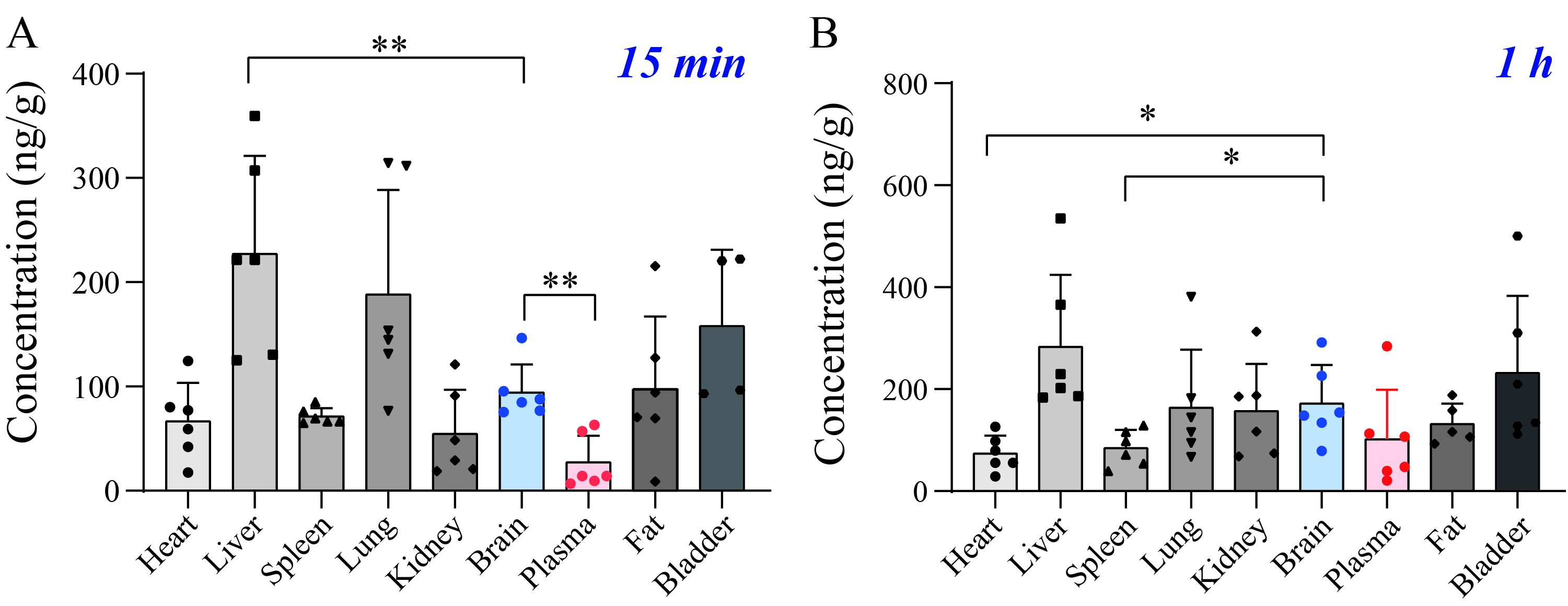


**Figure S4: Drug concentration in various tissues of rats at different time points after intragastric administration of 10 mg/kg.** (A) Drug concentration in various tissues 15 minutes after oral administration. (B) Drug concentration in various tissues 1 hour after oral administration. (Mean ± SD; Compared with brain group: **P* < 0.05, ***P* < 0.01, ****P* < 0.001; Brown-Forsythe and Welch ANOVA TEST)

***1.5 The Activation Ability of QO-83 to Key Binding Site Mutants***

Based on the results of RMSD and molecular docking, we identified that the key binding sites between compound QO-83 and the K_V_7.2 channel are W236, and the binding with the C chain is more stable. Therefore, we conducted activity validation of point mutations at W236 and F104. We found that 10 µM QO-83 had no effect on the activation curve of K_V_7.2^W236L^, with the V_1/2_ of activation shifting from -18.91 ± 3.20 mV to -19.33 ± 2.09 mV, showing no significant change in V_1/2_. Similarly, there was no significant effect on the current density of K_V_7.2^W236L^ (**Figure S5A, S5E**). However, 10 µM QO-83 shifted the activation curve V_1/2_ of K_V_7.2^F104A^ from -17.06 ± 1.27 mV to -25.61 ± 1.08 mV, a hyperpolarizing shift of 8.55 mV (**Figure S5B**). Although there was a slight leftward shift in the activation curve, compared to K_V_7.2^WT^, 10 µM QO-83 induced a hyperpolarizing shift of 26.38 mV in V_1/2_, indicating that the F104 mutation reduced the ability of QO-83 to open the K_V_7.2 channel. Meanwhile, QO-83 did not have a significant effect on the current density of K_V_7.2^F104A^ (**Figure S5F**). The reason might be that the W236 allele and others still have binding activity, and the mutation of F104 does not completely eliminate this binding. Therefore, W236 is a key binding site for QO-83 with the K_V_7.2 channel, while F104 also plays an important role in the binding interaction.

In addition, we validated two K_V_7.2 mutation sites, Y284 and A306, which are highly associated with benign familial neonatal epilepsy. Both mutations occur in the pore region of the K_V_7.2 channel. RTG has been shown to have a weak interaction with the K_V_7.2^A306^ residue and can also promote the opening of K_V_7.2^Y284^ currents. Therefore, we verified the effect of QO-83 on the mutant currents at these two sites. We found that 10 µM QO-83 shifted the activation curve V_1/2_ of K_V_7.2^Y284C^ from -18.46 ± 3.20 mV to -28.04 ± 2.81 mV, a hyperpolarizing shift of 9.58 mV, which was smaller than the shift observed in K_V_7.2^WT^ (**Figure S5C**). Furthermore, 10 µM QO-83 still increased the current density of the K_V_7.2^Y284C^ channel under 0 mV, 1000 ms stimulation (**Figure S5G**). Similarly, 10 µM QO-83 shifted the activation curve V_1/2_ of K_V_7.2^A306T^ from -27.58 ± 1.23 mV to -44.44 ± 0.90 mV, a hyperpolarizing shift of 16.86 mV, and also significantly increased the current density (**Figure S5D, S5H**). Therefore, although the K_V_7.2^Y284C^ and K_V_7.2^A306T^ mutations retain some activity in terms of activation and increasing current, QO-83 may still have therapeutic potential for epilepsy related to these mutation sites.


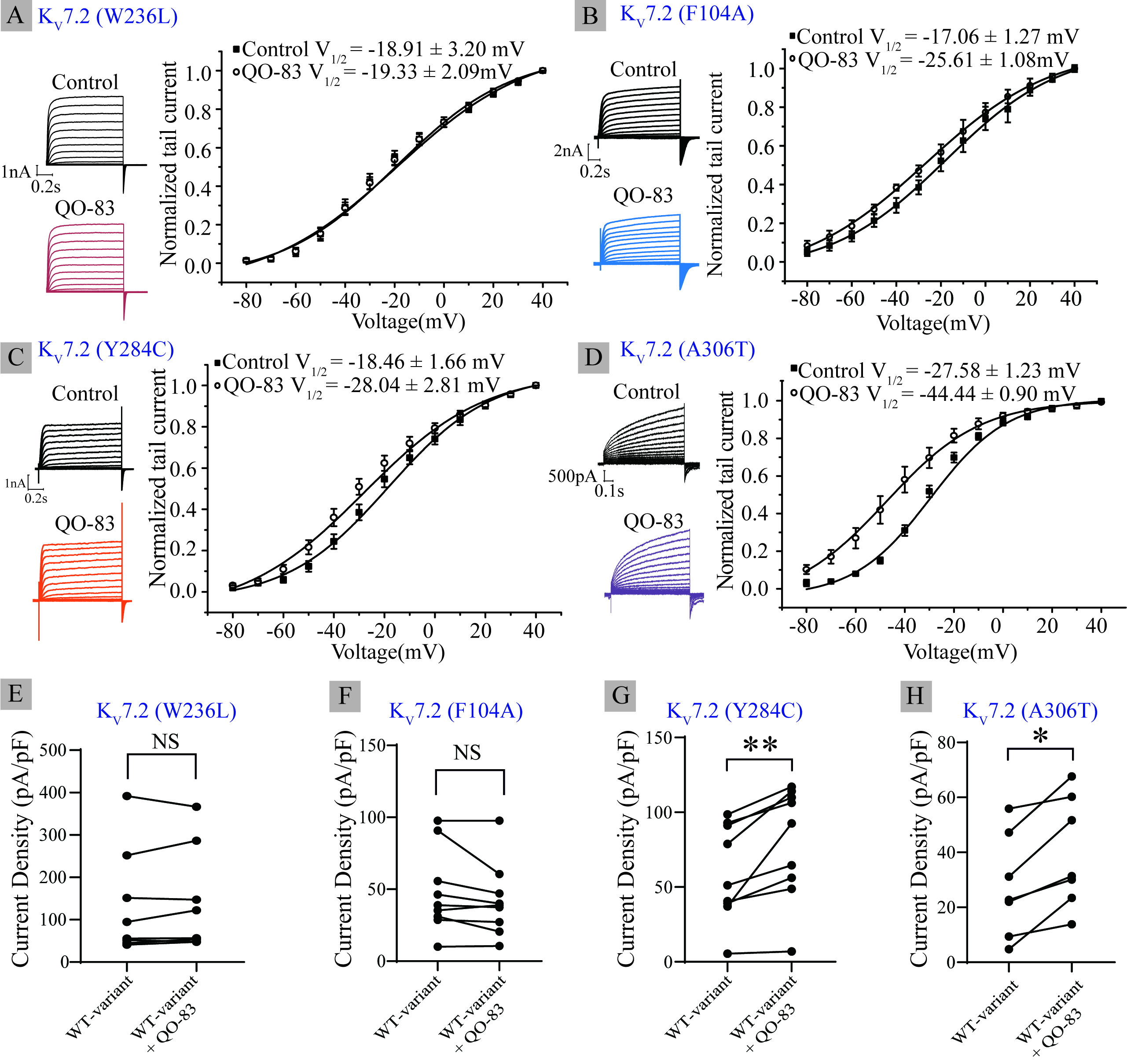


**Figure S5: The effect of QO-83 on activation curve of K_V_7.2^W236L^, K_V_7.2^F104A^, K_V_7.2^A306T^ and K_V_7.2^Y284C^ channels.** (A)The effect of QO-83 on the activation curve of K_V_7.2^W236L^ (n = 6). (B)The effect of QO-83 on the activation curve of K_V_7.2^F104A^(n = 8). (C)The effect of QO-83 on the activation curve of K_V_7.2^Y284C^ (n = 6). (D)The effect of QO-83 on the activation curve of K_V_7.2^A306T^ (n = 6). (E-H)The effect of 10 µM QO-83 on the current density of K_V_7.2^W236L^, K_V_7.2^F104A^, K_V_7.2^A306T^ and K_V_7.2^Y284C^ channels under 0 mV, 1000 ms stimulation. (**P* < 0.05, ***P* < 0.01, ****P* < 0.001; Shapiro-Wilk test and paired sample t-test)

***1.6 Stability Evaluation of QO-83***

The dimerization of RTG is known to cause the development of a blue-purple precipitate due to its structural instability, coupled to melanin playing a role in this process. To evaluate the stability of RTG and compound QO-83, we conducted in vitro tests to determine whether QO-83 undergoes polymerization. Both drugs were dissolved in saline at a concentration of 10 μM and maintained at a constant temperature of 37°C for 30 days. Color changes in the drug solutions were observed at room temperature. Compound QO-83 exhibited no noticeable color changes, whereas the RTG solution developed a distinct purple coloration (**Figure S6**).

Using liquid chromatography-mass spectrometry (LC-MS), the contents of RTG and QO-83 in a 20% β-cyclodextrin solution were analyzed over 30 days. The RTG content significantly decreased in the presence and absence of melanin (**Figures S7-S8**). LC-MS detected and identified RTG dimers with a molecular weight of 601.3 (**Figure S9**). Peak areas of RTG and QO-83, both individually and in mixtures with melanin, were integrated for quantitative analysis after 1, 10, 20, and 30 days. The content of RTG showed a significant decrease, whereas QO-83 exhibited no significant changes (**Figures S7-S8**). In conclusion, both the appearance of the drug solution and content analysis demonstrated that compound QO-83 exhibits greater chemical stability than RTG.

**
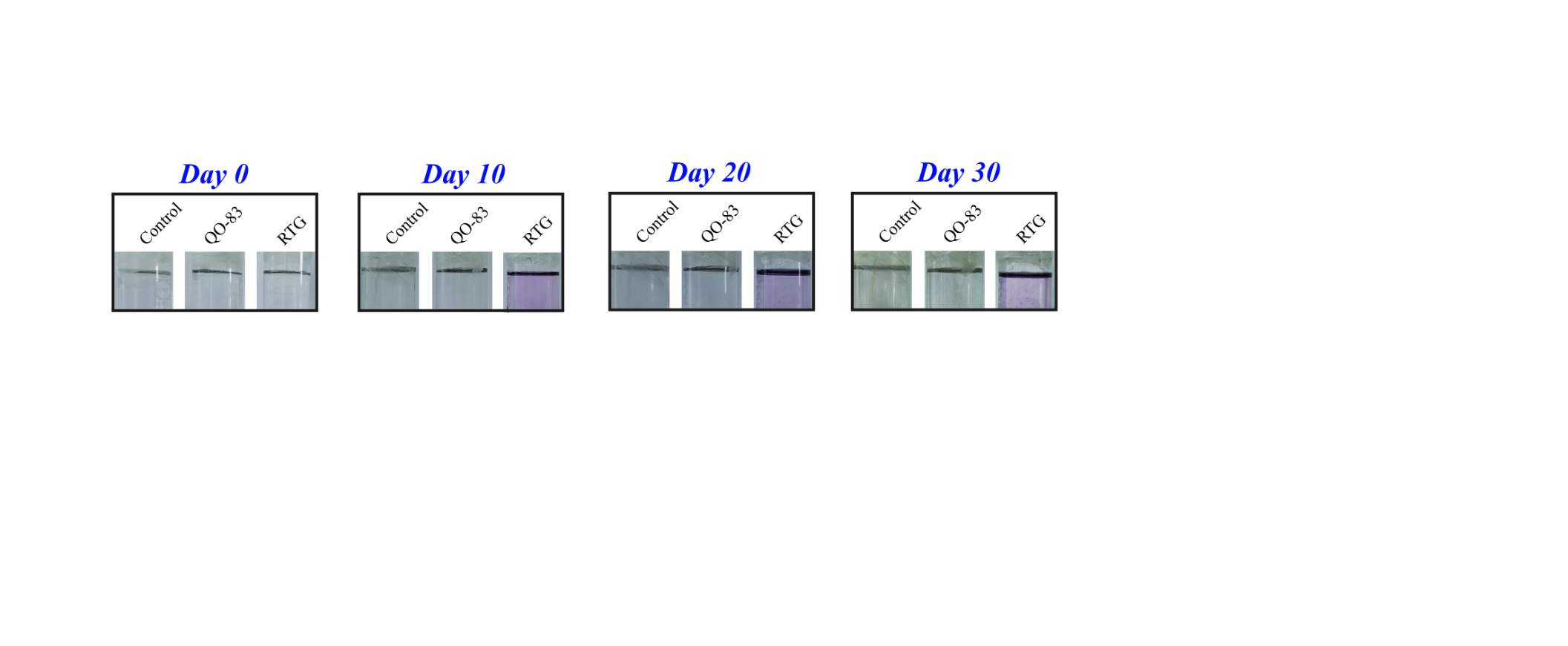
Figure S6: Time-dependent color changes of solutions containing compound QO-83 and RTG.**

**
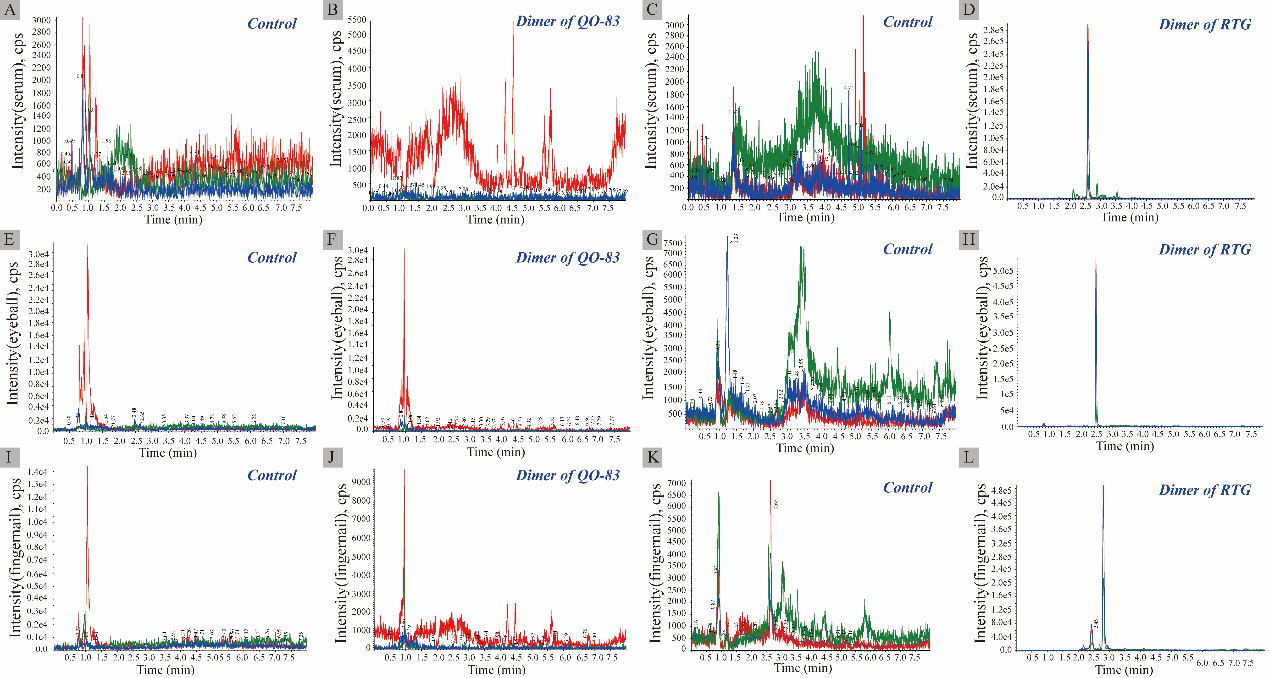
Figure S7: Detection of compound QO-83 dimerization in vivo using LC-MS/MS.** (A) Typical extracted ion chromatogram of a blank control group of the compound QO-83 dimer in blood. (B) Typical extracted ion chromatogram of the QO-83 dimer in blood. (C) Typical extracted ion chromatogram of a blank control group of RTG dimer in blood. (D) Typical extracted ion chromatogram of RTG dimer in blood. (E) Typical extracted ion chromatogram of a blank control group of the QO-83 dimer in the eyeballs. (F) Typical extracted ion chromatogram of the QO-83 dimer in the eyeballs. (G) Typical extracted ion chromatogram of a blank control group of RTG dimers in the eyeballs. (H) Typical extracted ion chromatogram of RTG dimer in the eyeballs. (I) Typical extracted ion chromatogram of a blank control group of the QO-83 dimer in fingernail. (J) Typical extracted ion chromatogram of dimer of QO-83 in fingernail. (K) Typical extracted ion chromatogram of a blank control group of RTG dimer in fingernail. (L) Typical extracted ion chromatography of RTG dimer in fingernail.


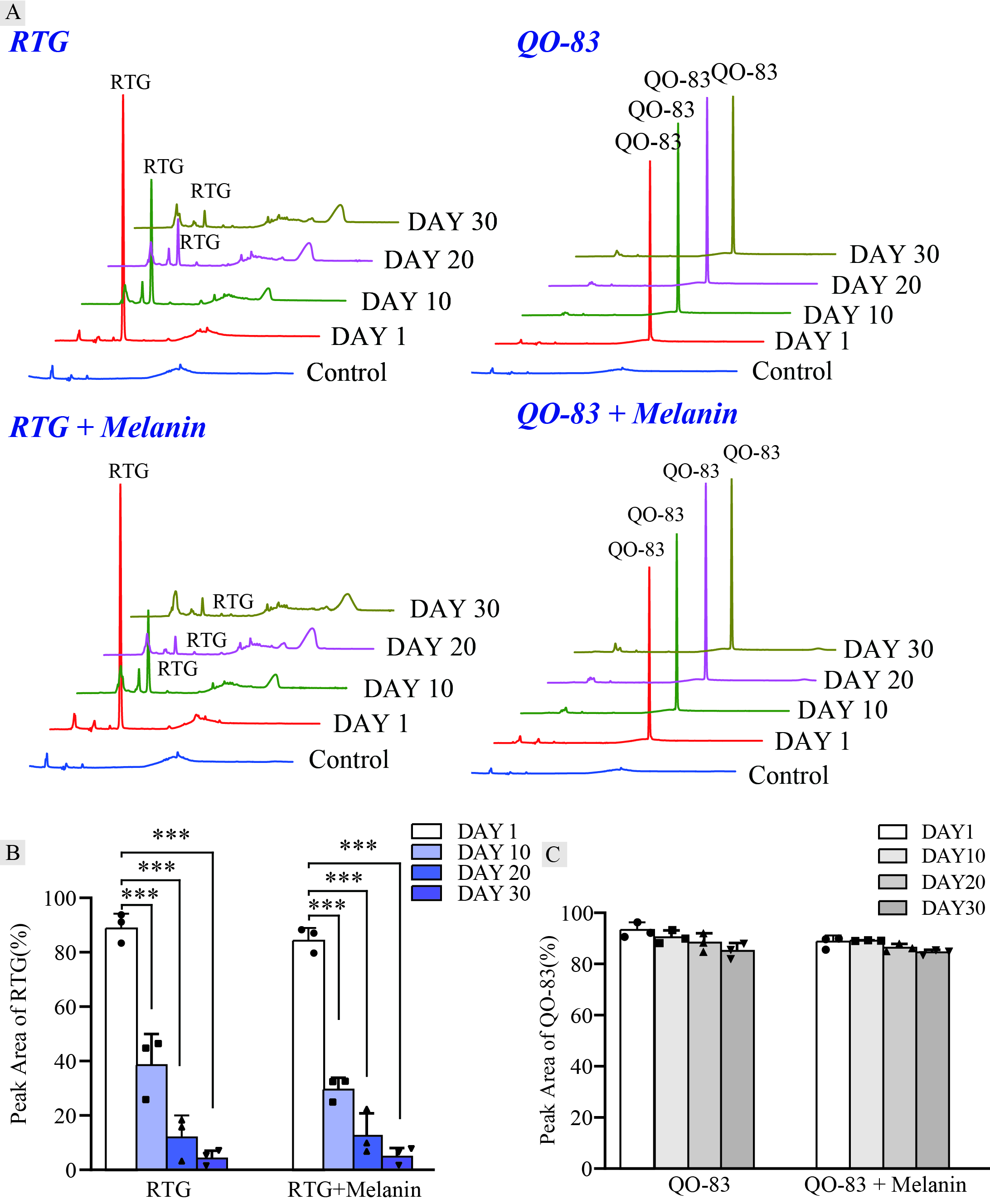


**Figure S8: Stability experiment of compound QO-83.** (A) Typical liquid chromatogram of QO-83 and RTG at different time points. (B) Columnar statistical plot of the peak area from the liquid chromatogram for the RTG and RTG + melanin groups (n = 3). (C) Columnar statistical plot of the peak area from the liquid chromatogram for the QO-83 and QO-83 + melanin groups (n = 3). (**P* < 0.05, ***P* < 0.01, ****P* < 0.001; Bonferroni–ANOVA test).

**
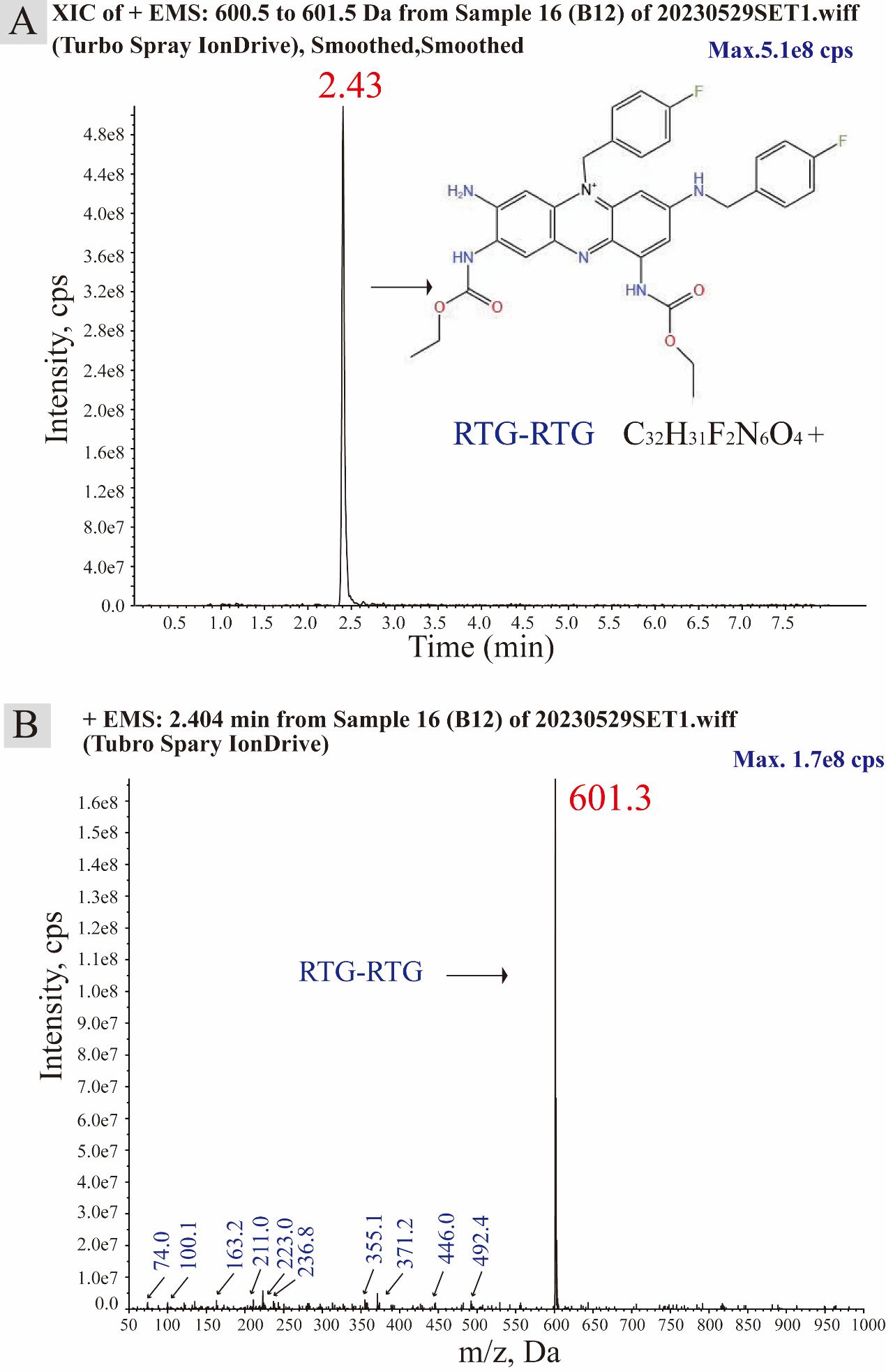
**

**Figure S9: RTG dimer structural identification using liquid chromatography-mass spectrometry (LC-MS).** (A) The extracted ion chromatogram of RTG dimer (RTG-RTG) (n=3). (B) The mass spectrogram of RTG dimer (RTG-RTG) (n=3)**.**

***1.7*** ***Toxic effects of QO-83 on the liver***

The control group exhibited no pathological alterations in hepatic lobule structure. Hepatocyte distribution appeared cord-like, with no abnormal changes in cell volume or nuclear integrity. Fibrous connective tissue proliferation or inflammatory cell infiltration was absent in hepatic lobules and portal veins (**Figure S10A**). Conversely, the Acetaminophen (APAP) group showed significant pathological changes, including enlarged hepatocytes, altered nuclear positions, and severe diffuse granular degeneration (**Figure S10A**).Notably, the 20mg/kg QO-83 group exhibited no discernible abnormalities in liver tissues. Hepatocytes maintained structural integrity, arranged radially around the central vein within hepatic lobules (**Figure S10A**).

Compared to the control group, the liver-body weight ratio increased significantly in the APAP, Flupirtine, and QO-83 groups (**Figure S10B**). However, among these, 20mg/kg QO-83 showed the least increase in liver weight ratio. Body weight did not significantly differ among the administration groups (**Figure S10C**). MTT experiments revealed that incubating L02 cells with 0.1-100 μM QO-83 for 24h and 48h had no significant impact on cell viability (**Figure S10D, S10E**). In contrast, exposure to 100 μM Flupirtine for the same duration significantly inhibited cell viability (**P* < 0.05, ****P* < 0.001) (**Figure S10D, S10E**).


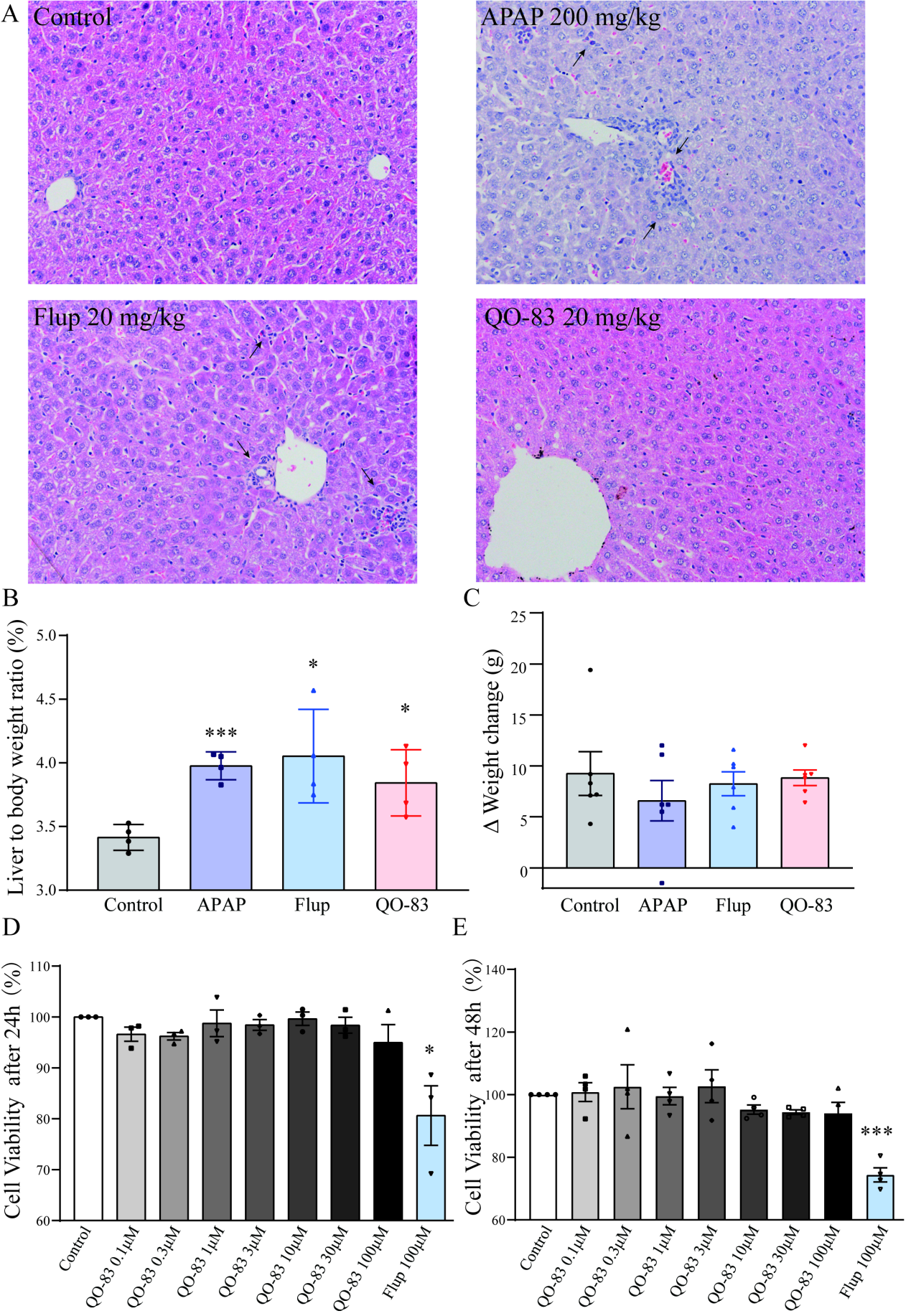


**Figure S10 Toxic effects of QO-83 on the liver.** (A) Representative images of HE staining pathological sections in each drug group (n = 4). (B) Histogram showing the liver weight ratio in each drug group (n = 4). (C) Bar chart depicting weight differences among drug groups (n = 4). (D) Histogram of cell viability after 24 hours of incubation with QO-83 (n = 4). (E) Histogram of cell viability after 48 hours of incubation with QO-83 (n = 4). (**P* < 0.05, ****P* < 0.001; Bonferroni–ANOVA test)

**Table S1 Effects of Compound QO-83 at different concentrations on the activation curves (V_1/2_) of various subtypes of K_V_7 channels. (mV)**

| Group | K_V_7.2 | K_V_7.2/7.3 | K_V_7.3 | K_V_7.4 | K_V_7.5 |
| --- | --- | --- | --- | --- | --- |
| Control | -18.99 ± 1.27 | -13.26 ± 1.60 | -25.24 ±1.30 | -22.51 ± 1.50 | -39.18 ± 1.90 |
| 0.01 μM QO-83 | -20.30 ± 1.48 | -13.15 ± 1.63 | -27.52 ± 1.41 | -23.98 ± 1.51 | -40.14 ± 1.96 |
| 0.05 μM QO-83 | -21.38 ± 1.03 | -13.35 ± 2.97 | -29.39 ± 1.57 | -24.11 ± 1.51 | -37.74 ± 1.98 |
| 0.10 μM QO-83 | -29.20 ± 1.24 | -13.29 ± 2.95 | -30.92 ± 1.23 | -25.55 ± 2.24 | -44.84 ± 2.17 |
| 0.50 μM QO-83 | -39.26 ± 1.00 | -17.90 ± 1.63 | -28.78 ± 1.67 | -27.22 ± 2.43 | -44.43 ± 3.78 |
| 1.00 μM QO-83 | -34.18 ± 1.18 | -25.70 ± 1.50 | -50.84 ± 1.015 | -35.87 ± 1.99 | -58.71 ± 2.45 |
| 5.00 μM QO-83 | -38.46 ± 1.19 | -36.05 ± 1.83 | -56.12 ± 1.01 | -35.78 ± 2.41 | -61.54 ± 2.70 |
| 10.00 μM QO-83 | -45.37 ± 1.26 | -41.05 ± 3.24 | -62.91 ± 1.18 | -32.52 ± 2.52 | -65.46 ± 2.19 |
| 10.00 μM RTG | -32.23 ± 1.89 | -37.76 ± 2.34 | -55.64 ± 1.31 | -40.62 ± 2.00 | -56.77 ± 1.07 |

**Table S2 The effects of QO-83 on channels activation and deactivation of Kv7 subtypes**

|  | Time constants (ms) | Control | QO-83(10 μM) | RTG(10 μM) |
| --- | --- | --- | --- | --- |
| K_V_7.2 | Activation τ (10 mV) | 210.30 ± 42.56 | 201.23 ± 62.21 | 259.60 ± 69.06 |
|  | Deactivation τ (-120 mV) | 48.13 ± 3.81 | 115.04 ± 18.26* | 102.04 ± 18.32* |
| K_V_7.2/7.3 | Activation τ (10 mV) | 159.73 ± 40.38 | 190.06 ± 32.38 | 205.66 ± 37.03 |
|  | Deactivation τ (-120 mV) | 61.98 ± 2.32 | 173.70 ± 40.16* | 154.46 ± 45.81* |
| K_V_7.3 | Activation τ (10 mV) | 209.72 ± 24.10 | 221.50 ± 32.08 | 246.33 ± 21.28 |
|  | Deactivation τ (-120 mV) | 41.16 ± 3.07 | 125.31 ± 15.77* | 153.33 ± 48.97* |
| K_V_7.4 | Activation τ (10 mV) | 214.75 ± 30.96 | 227.33 ± 52.03 | 242.74 ± 34.66 |
|  | Deactivation τ (-120 mV) | 35.41 ± 6.40 | 72.08 ± 6.24* | 99.62 ± 18.87* |
| K_V_7.5 | Activation τ (10 mV) | 155.33 ± 14.63 | 181.42 ± 22.22 | 170.33 ± 16.42 |
|  | Deactivation τ (-120 mV) | 58.61 ± 5.54 | 95.34 ± 8.82* | 96.4 ± 14.98* |

(The data were represented as Mean ± SE; n = 6 ~ 12; *:compare with control; **P* < 0.05; LSD-ANOVA test )

**2 Materials and Methods**

***2.1 Stability of QO-83 was tested by High Performance Liquid Chromatography (HPLC) and Mass Spectrometry (MS) Detection***

The stability assessment of QO-83 was conducted using High-Performance Liquid Chromatography (HPLC) coupled with Mass Spectrometry (MS) detection. Experimental groups included a control group containing 20% β-cyclodextrin, a 100 μM group treated with RTG (Shanghai Adama Reagent Co., LTD.), a 100 μM group treated with QO-83, and groups treated with combinations of RTG or QO-83 with melanin (50 μM) from Melanin sigma Co., USA. These groups were incubated in a constant temperature environment of 37℃ for 30 days, with sample collection performed on days 1, 10, 20, and 30 post-preparation.

For sample preparation, collected samples were diluted with pure methanol at a 1:1 ratio. The resulting mixture underwent centrifugation at 13,000 rpm for 15 minutes, after which the supernatant was extracted for HPLC analysis. The HPLC analysis utilized an Agilent 1200 liquid chromatography system equipped with a Pinnacle DBC18 5 μm (250×4.6 mm) chromatographic column.

The gradient program spanned specific time intervals: 0 to 5 minutes utilized a mixture of water and methanol at a ratio of 38:62, followed by a transition to a 15:85 water-methanol ratio from 5 to 7 minutes, and maintained at 15:85 for the duration of 7 to 20 minutes. The system then ran for an additional 20 minutes. A consistent flow rate of 1 ml/min was maintained throughout the analysis. Detection was accomplished at a wavelength of 253 nm, while the column temperature was rigorously controlled at 40°C, with 20 μl of the sample injected for each analysis.

For the detection and quantification of potential polymers, sophisticated mass spectrometry techniques were employed using advanced instruments. Specifically, a 4000 Q-Trap mass spectrometer was utilized in enhanced parent scan mode, alongside an SCIEX QTRAP 6500 mass spectrometer operating in multiple reaction detection mode. Key parameters for the mass spectrometry analysis included an electrospray voltage (IS) of +5500 V, ion source temperature (TEM) set at 450℃, curtain gas (CUR) at 20 psi, atomizing gas (GS1) at 45 psi, and auxiliary gas (GS2) at 50 psi. Chromatographic separation was achieved using a Phenomenex Kinetex XB-C18 chromatographic column (2.6 μm, 100×3 mm) at a flow rate of 0.4 ml/min. The mobile phase consisted of a prepared mixture of methanol and a 0.1% solution of formic acid, optimized for effective separation and detection. Samples were appropriately diluted tenfold, with 5 μl of the diluted sample injected into the system for analysis, ensuring accurate quantification and characterization of potential polymers generated during the experimental procedures.

***2.2 Cell Culture and High-throughput Screening of QO-83 for Sensitivity to K_V_7 Channels***

*2.2.1 Cell Culture*

Stable Chinese Hamster Ovary (CHO) cell lines expressing specific Kv7 channel subtypes, including K_V_7.2 and K_V_7.2/7.3, were cultured in Minimum Essential Medium (MEM) supplemented with 10% fetal calf serum, 1× nonessential amino acids, 600 mg/mL G418, and 600 mg/mL hygromycin B. Concurrently, K_V_7.4 and K_V_7.5 channels were stably expressed in Human Embryonic Kidney 293A (HEK293A) cell lines and nurtured in Dulbecco's Modified Eagle Medium (DMEM) high glucose medium supplemented with 10% fetal bovine serum (FBS), 600 mg/mL G418, and 600 mg/ml hygromycin B.

*2.2.2* *ICR 8000 rubidium ion elution high-throughput screening experiment detects the sensitivity of QO-83 to K_V_7.2/7.3, K_V_7.4 and Kv7.1 channels.*

The high-throughput screening experiment utilizing the ICR 8000 rubidium ion elution method aimed to assess the sensitivity of QO-83 towards K_V_7.2/3 and K_V_7.1 channels. Cells in the logarithmic growth phase were seeded into 96-well plates at a density of 2×105 cells per well. Following overnight incubation, the medium was replaced with 200 μL of RBCL-loaded buffer and incubated under controlled conditions (5% CO_2_, 37°C) for 3 hours. The loading buffer composition consisted of 5.4 mM RbCl, 5 mM Glucose, 25 mM HEPES, 150 mM NaCl, 1 mM MgCl_2_, 0.8 mM NaH_2_PO_4_•2H_2_O, and 2 mM CaCl_2_.

Subsequently, the solution was aspirated, and the cells were washed thrice with Washing Buffer (5.4 mM KCl, 25 mM HEPES, 150 mM NaCl, 1 mM MgCl_2_, 0.8 mM NaH_2_PO_4_•2H_2_O, 2 mM CaCl_2_). Positive control drug RTG and compound QO-83 were then diluted in Open Buffer (20 mM KCl, 25 mM HEPES, 130 mM NaCl, 1 mM MgCl_2_, 0.8 mM NaH_2_PO_4_•2H_2_O, 2 mM CaCl_2_, pH 7.4 adjusted with NaOH) to establish seven concentration gradients (100, 30, 10, 3, 1, 0.3, and 0.1 µM). Each concentration was distributed across triplicate wells, with corresponding concentration solvent control wells included. Incubation of cells with 200 µL of the test solutions followed, allowing for a 10-minute reaction period within a 37°C, 5% CO_2_ incubator.

Post-incubation, 200 µL of the reaction supernatant was carefully transferred to a new 96-well plate for Rb+ absorption measurement at 780 nm using an ICR 8000 atomic absorption analyzer. Instrument parameters were set, including Average Current (8 mA), Entrance Slit (0.6 nm), Peak Wavelength (Auto gain), Integration time (10 s), Delay (0 s), and Autozero at regular intervals. Each injection volume was maintained at 100 µL, ensuring consistency and accuracy throughout the experimental procedure.

***2.3 HPLC-MS/MS detection of drug concentrations of compound QO-83 in various tissues of SD rats.***

Eighteen male SD rats were randomly divided into three groups, with six rats in each group. After grouping, the rats were kept under normal conditions for three days, fasted for 12 hours prior to the experiment, and provided free access to water. The rats were administered intragastrically, meaning they received doses of 10 mL/kg (10 mg/kg) body weight. After intragastric administration, blood samples were collected at 15 min and 2 h. Blood was collected from the orbital venous plexus using heparinized centrifuge tubes. Before centrifugation, the collected blood was placed in an ice bath and centrifuged at 4500 rpm for 10 minutes at 4°C. The plasma was separated and stored at -40 °C. A precise 100 µL plasma sample was put into a 1.5 mL centrifuge tube. Then, 300 µL of methanol solution with an internal standard (20 ng/mL) was added. The mixture was vortexed for 1 minute and centrifuged at 12,000 rpm for 10 minutes. Then, 100µL of the supernatant was transferred to another 1.5 mL centrifuge tube, and 200µL of the mobile phase was added. After vortexing for 20 seconds, 100 µL of the solution was transferred to an autosampler vial for HPLC-MS/MS analysis.

***2.4 Transfection of KCNQ2^F104A^/KCNQ2^W236L^/KCNQ2^Y284C^/KCNQ2^A306T^ mutant plasmids***

The CHO stable cell line was used for transfection, and the cells were cultured to 60%–80% confluence before the experiment. Prior to use, the transfection reagent (FuGENE HD, promega,USA ) was brought to room temperature, and the plasmid carrying the reporter gene or fluorescent protein (F104A/W236L/Y284C/A306T) was placed in sterile, serum-free medium to prepare the mixture of plasmid and transfection reagent. FuGENE HD was added directly to the medium containing the plasmid and incubated for 15 minutes. Electrophysiological recordings were performed 24 hours post-transfection, and all patch-clamp recording methods were conducted as described in Section 2.1. The protocol for recording current density involved holding at -120 mV, with a stimulus voltage of 0 mV for 1000 ms.

***2.5 Toxic effects of QO-83 on the liver***

*2.5.1 Effect of QO-83 on the liver tissue of KM mice.*

Twenty-four KM mice, half male and half female, weighing between 18 and 22 g, were randomly divided into four groups, each consisting of six mice: a control group, an acetaminophen (APAP) group, a flupirtine (FM) group, and a QO-83 group. The treatment lasted for 28 days, and the drug solvent was a 0.8% sodium carboxymethyl cellulose solution. The control group received an equal amount of 0.8% sodium carboxymethyl cellulose solution. A single dose was administered to each group via gavage, and the dosage volume was calculated based on the body weight of the mice. The control group received 0.8% sodium carboxymethyl cellulose solution, the APAP group received acetaminophen at 200 mg/kg/d, the FM group received fluorofenidone at 20 mg/kg/d, and the QO-83 group received QO-83 at 20 mg/kg/d. After 28 days of administration, the liver was isolated, fixed in 4% paraformaldehyde for at least 24 h, routinely embedded in paraffin, and sectioned into 3–5 μm continuous slices. Hematoxylin-eosin (HE) staining was performed to observe the pathological structure of the tissue. After staining with HE, the tissue was dehydrated using an alcohol gradient, cleared, and mounted for the observation of liver lesions under a light microscope.

*2.5.2 Evaluation of the cytotoxic effects of QO-83 in vitro.*

Normal human liver L02 cells were cultured in RPMI-1640 complete medium and maintained in a humidified incubator at 37°C with 5% CO_2_. Cell status was observed daily, and the medium was changed every 1–2 days. During the experiment, cells in good condition and in the logarithmic growth phase were selected. After digestion and collection, the cells were evenly suspended, and the cell suspension concentration was adjusted to 5×10^4^ cells/mL. A volume of 150 μL was inoculated per well in a 96-well plate, leaving the surrounding wells for the addition of sterilized PBS (100 μL per well) to prevent edge effects. The plate was then placed in an incubator for further incubation. After 12 h of culturing to allow cell adhesion, the culture medium was aspirated from the 96-well plate. Based on the control, QO-83 was added at concentrations of 0.1 μM, 0.3 μM, 1 μM, 3 μM, 10 μM, 30 μM, and 100 μM, along with Flupirtine (100 μM) in a concentration gradient of 150 μL per well, with six replicates for each group. The control group served as the normal control. The cultures were incubated for 24 and 48 h, respectively. After this period, the culture medium was removed, and 50 μL of 5 mg/mL MTT solution was added to each well, followed by continued incubation for 4 h. In a light-protected environment, the 96-well plate was removed, and the culture medium was carefully aspirated from the wells. Then, 200 μL of DMSO was added to each experimental sample well. To ensure complete dissolution of the crystals, the 96-well plate was shaken at low speed on a shaker for 10 min. Finally, the absorbance was measured using a microplate reader (preheated for at least 15 min) at a wavelength of 490 nm.
